# Extra Large G-Protein Interactome Reveals Multiple Stress Response Function and Partner-Dependent XLG Subcellular Localization

**DOI:** 10.3389/fpls.2017.01015

**Published:** 2017-06-13

**Authors:** Ying Liang, Yajun Gao, Alan M. Jones

**Affiliations:** ^1^College of Natural Resources and Environment, Northwest A&F UniversityXianyang, China; ^2^Department of Biology University of North Carolina at Chapel HillChapel Hill, NC, United States; ^3^Department of Pharmacology, University of North Carolina at Chapel HillChapel Hill, NC, United States

**Keywords:** Arabidopsis, extra-large G protein, XLG protein interactome, salt stress, SZF, yeast two hybrid, NaCl

## Abstract

The three-member family of Arabidopsis extra-large G proteins (XLG1-3) defines the prototype of an atypical Gα subunit in the heterotrimeric G protein complex. Recent evidence indicate that XLG subunits operate along with its Gβγ dimer in root morphology, stress responsiveness, and cytokinin induced development, however downstream targets of activated XLG proteins in the stress pathways are rarely known. To assemble a set of candidate XLG-targeted proteins, a yeast two-hybrid complementation-based screen was performed using XLG protein baits to query interactions between XLG and partner protein found in glucose-treated seedlings, roots, and Arabidopsis cells in culture. Seventy two interactors were identified and >60% of a test set displayed *in vivo* interaction with XLG proteins. Gene co-expression analysis shows that >70% of the interactors are positively correlated with the corresponding XLG partners. Gene Ontology enrichment for all the candidates indicates stress responses and posits a molecular mechanism involving a specific set of transcription factor partners to XLG. Genes encoding two of these transcription factors, SZF1 and 2, require XLG proteins for full NaCl-induced expression. The subcellular localization of the XLG proteins in the nucleus, endosome, and plasma membrane is dependent on the specific interacting partner.

## Introduction

In animals, the heterotrimeric G protein complex consists of Gα, Gβ, and Gγ subunits and is tethered to the cytoplasmic side of the plasma membrane nestled with 7 transmembrane (Hamm, 1998; Oldham and Hamm, 2008). G protein coupled receptors (GPCR). GPCRs receive extracellular signals and then activate the G protein signaling pathway by catalyzing GDP removal from the Gα subunit allowing GTP binding and subsequent release of the Gβγ dimer (Wettschureck, 2005; Li et al., 2007; Oldham and Hamm, 2008). The Gα subunit has an intrinsic GTP hydrolysis rate that returns the heterotrimeric complex to its basal (resting) state. Regulator of G Signaling (RGS) proteins accelerate GTP hydrolysis. The human genome encodes 23 Gα, 5 Gβ, and 12 Gγ subunits, ~850 GPCRs, and ~40 RGS proteins. In Arabidopsis, the heterotrimeric G protein complex consist of one canonical alpha subunit (AtGPA1), one beta subunit (*AGB1*), one of three gamma subunits (AGG1, 2, and 3), at least one subunit of Regulator of G Signaling protein (AtRGS1), and one of three atypical Extra-Large G proteins (XLG1, 2, and 3) (Pandey et al., 2006; Ding et al., 2008; Chakravorty et al., 2011; Urano et al., 2013, 2016; Wolfenstetter et al., 2015) in lieu of the canonical G subunit. In plants, the mechanism for activation is different than in animals; the Gα subunit self-activates without a GPCR and instead is kept in the basal state by a 7 transmembrane RGS protein (Urano et al., 2012, 2013).

The presence of atypical XLGs makes G protein signaling in plants unique. The primary sequence conservation of the C-terminal Gα domain of the three XLG proteins compared to the canonical Gα are 26.1, 23.2, and 28.5% identities for XLG1^446–888^, XLG2^435–861^, and XLG3^396–848^, respectively (Ding et al., 2008; Chakravorty et al., 2015; Urano et al., 2016). The Gα domain of XLGs is structurally similar to AtGPA1 containing three of five G-box motifs that are critical for binding the guanine nucleotide. The N-terminal region of XLGs contains a nuclear localization signal (NLS) and at least XLG3 in this family encodes a functional nuclear export signal (NES) (Chakravorty et al., 2015). Whether or not XLGs bind guanine nucleotides is unclear but the evidence to date indicate that if they do, the mode is different from the canonical Gα subunit (Lee and Assmann, 1999; Heo et al., 2012). *In vitro* studies indicate that XLGs bind the Gβγ dimer but do so unlike the canonical Gα subunit (Maruta et al., 2015) and possibly do so independently of nucleotide binding (Urano et al., 2016). Finally, there is uncertainty in the literature about the subcellular location of XLGs. Ding et al. (2008) found fluorescence localized to the nucleus when XLGs-GFP or the N-terminal XLG-GFP proteins were transiently overexpressed in *Vicia faba* guard cells, while Maruta et al. (2015) found that the XLG proteins are located in both nucleus and plasma membrane (XLG1-GFP only displayed a plasma membrane signal) in the Arabidopsis stable transgenic lines with GFP-XLGs, however when in complex with the Gβγ dimer, XLGs are only located on the plasma membrane (Maruta et al., 2015). Chakravorty et al. (2015) confirmed the interaction of the XLG with the Gβγ dimer at the plasma membrane but also showed that when the XLG-GFP protein was not obligated to partner with the Gβγ dimer, fluorescence was noted in the nucleus. These findings raise the possibility that XLG subcellular localization is conditional.

It is established that G proteins are involved in various stress responses. AtRGS1 is a glucose sensor (Grigston et al., 2008), but is also involved in regulating certain stress responses. For example, the null mutants of AtRGS1 are more resistant to salt stress (Colaneri et al., 2014). The AGB1 mutants are hypersensitive to salt stress (Yu and Assmann, 2015), ER stress, and glucose stress (Pandey et al., 2006). Similarly to the AGB1 mutants, the XLG1/2/3 triple mutant is hypersensitive to salt, tunicamycin, and D-glucose in post germination development (Chakravorty et al., 2015; Maruta et al., 2015; Urano et al., 2016). XLG proteins are involved in stress responses (Urano et al., 2016), however, the mechanism is unclear.

Few protein partners to XLGs are known. Heo et al. (2012) performed a yeast two hybrid (Y2H) screen for XLG2 and found that XLG2 interacts with the nuclear protein RTV1 (related to vernalization 1). Wang et al. (2017) identified two plant U-box E3 ligases (PUB2 and 4) that interact with XLG proteins. The double *pub1/2* mutant shares developmental phenotypes to the *xlg1/2/3* triple mutant. These two groups did not report any other XLG interactor.

In order to elucidate the mechanism for XLG-mediated stress responsiveness and development, we must first assemble the set of proteins operating in the associated pathways. Here, we report a set of proteins identified in a Y2H screen for XLG protein partners. A large proportion of the identified candidate XLG interactors are implicated in various stress responsive signaling pathways. Many of these are confirmed to interact with XLG *in vivo*. Analyses of the consequences of XLG-partner interaction revealed that the subcellular localization of the XLG protein is conditional on its binding partner, resolving the conflicting published data on XLG localization.

## Materials and methods

### Yeast two hybrid screening for the XLG interactome

The process of screening the interactome of XLGs using Y2H followed the Yeast Protocols Handbook by Clontech. Briefly, the full length XLGs were cloned into the pENTR/D-TOPO vector (Invitrogen), then recombined into the pAS2-1 GATEWAY vector (Criekinge and Beyaert, 1999). The bait vectors were transformed into *Saccharomyces cerevisiae* strain AH109 and autoactivation was tested in the triple dropout plates (-Trp-His-Ade) with the indicated proper amounts of 3-amino-1,2,4-triazole (3-AT). The library plasmids were transformed into the Y187 strain according to the “Mate & Plate™” Library System User Manual from Clontech (http://www.clontech.com). All media and reagents were made as indicated by the manufacturer (Clontech). After mating, the culture was spread on dropout (DO) plates according to the autoactivation results as follows. For XLG1, the first screen was on quadruple DO (-Try/Leu/His/Ade) plates with 10 mM 3-AT and subsequently screened on quadruple DO plates with 20 mM 3-AT. For XLG2 and XLG3, transformants were spread on triple DO (-Try/Leu/His) plates with 1 mM 3-AT and subsequently screened on quadruple DO (-Try/Leu/His/Ade) plates with X-alpha-gal. Positive colonies further purified by spreading on new plates twice for confirmation and then cultured in –Leu liquid medium to rescue the plasmids. Plasmid DNA were amplified with primer GAL4-AD-Fw (AATACCACTACAATGGAT). Plasmids were restricted with enzymes *Hind*III, *Ava*I, and *Sma*I individually to eliminate duplicates before sequencing. The sequencing results were analyzed by BLAST in NCBI (https://www.ncbi.nlm.nih.gov/).

### Genetic stocks

The following T-DNA insertion mutants were used: *agb1-2* (Ullah et al., 2003), *rgs1-2* (Chen et al., 2003), *gpa1-3* (Jones et al., 2003), *xlg1xlg2xlg3* (Ding et al., 2008) which combines these alleles *xlg1-1* (SAIL_760H08) (Ding et al., 2008), *xlg2-2* (SALK_062645), *xlg3-2* (SAIL_107656) (Ding et al., 2008), and *xlg/gpa1* which combines the *xlg1-1, xlg2-1, xlg3-2*, and *gpa1-3 alleles* above (Urano et al., 2016). The ATG number for the genes are *At4g34460 (AGB1); At2g26300 (GPA1); At3g26090 (RGS1); At2g23460 (XLG1); At4g34390 (XLG2); At1g31930 (XLG3)*. The single mutants of *szf1* (Salk_002993, Salk_141550) and *szf2* (Salk_024800C, CS873730) were obtained from the Arabidopsis Biological Resource Center (ABRC, http://www.arabidopsis.org/) and made homozygous.

### BiFC

BiFC was performed as described by Klopffleisch et al. (2011) and Tunc-Ozdemir et al. (2016). Briefly, pENTR clones of the genes of interest were subcloned into pCL113_JO (for N-terminal tagged cYFP) and the bait genes were subcloned into pCL112 and pBatL-sYFP-C (for C-terminal tagged cYFP). A positive-transformation control [mitochondrial RFP marker; mt-rk obtained from the ABRC (CD3-991)] was used to distinguish gene silencing from lack of protein complementation. NLS-CFP were used as a nucleus marker for the subcellular localization. Leaf samples were imaged using a Zeiss LSM710 confocal laser scanning microscope equipped with an Apochromat X40 (NA 1.2) water immersion objective. YFP and RFP were excited by a 514-nm argon laser and a 560-nm diode laser, respectively, and their respective emissions were detected at 526–569 and 565–621 nm by a photomultiplier detector. The digital images were analyzed with Zen software (Zeiss). CFP was excited with a 458-nm argon laser and the emission was detected at 490 nm.

### Culture of arabidopsis and salt treatment

Arabidopsis seeds were sterilized with 70% ethanol for 10 min, 95% ethanol for 10 min and sterilized water wash at least three times. Liquid culture of Arabidopsis was described by Grigston et al. (2008). Briefly, sterilized seeds were stratified at 4°C in the dark for 3–4 days, and then transferred to 250-mL flasks with 100 mL¼ MS liquid medium with 1% sucrose at a density of about 50 seeds per flask. The seedlings were grown under constant dim light (35–50 μEm^−2^s^−1^) shaking at 120 rpm for 7 days. While performing the treatment, the seedlings were gently removed and transferred into new flasks with ¼ MS liquid medium plus the indicated concentrations of NaCl. After treatment, seedlings were gently dried on tissue and wrapped in aluminum foil before flash freezing with liquid nitrogen. Samples were stored at −80 C until analyses.

### RNA isolation, reverse transcription, and real time PCR

Total RNA was isolated from seedlings treated with the indicated NaCl treatments were frozen in liquid nitrogen followed by homogenization in a mortar. RNA extraction was performed using an RNeasy Mini Kit (QIAGEN; Cat No: 74106) according to manufacturer's instructions. Residual genomic DNA was removed by treatment with RNase free DNase I (Thermo Fisher; Cat No. AM2222). First Strand cDNA was synthesized from 5 ug of total RNA using Thermo Scientific Maxima Reverse Transcriptase (Cat No: EP0741) according to the suppliers protocol using an anchored oligo (d) T primer mix. Real time PCR was performed using DNA Engine Opticon 2 System from MJ research and the comparative Ct-value (Threshold Cycle defined as the cycle number at which the fluorescence generated within a reaction crosses the threshold line) was measured with SYBR green (Invitrogen). The relative gene expression level was expressed as 2^−ΔΔCt^ as described by Livak and Schmittgen (2001). Primers are provide in Supplementary Table S2. PCR- program steps were: 94 degree C for 5 min, [9°C for 10 s, 62°C for 20 s, 72°C for 20 s] 40 times followed by a dissociation curve measurement.

### Salt stress assay

The growth medium was ¼ MS medium with 1% sucrose, 1 g/L 2-(*N*-morpholino) ethanesulfonic acid and 0.8% phytoagar (pH5.7) with 5 M NaCl stock added to make the indicated NaCl concentrations. The seeds on plates were kept at 4°C for 3 days in the dark and then the plates were transferred to constant light for 14 days.

### Protoplast isolation and transformation

Protoplast transformation was performed as described (Yoo et al., 2007). Briefly, 5-week-old Arabidopsis plants grown under short days (8/16 h light/dark) were used for the protoplast isolation. After the transformation, protoplasts were incubated in 24-well-plates for 2 days and the samples were imaged as described above.

## Results and discussion

### XLG interactome

Full length XLGs proteins were used as baits to screen for interacting proteins using the Y2H assay. Tests for auto-activation by the baits indicated that XLG1 auto-activates however, optimization with 3-AT (3-amino-1,2,4-triazole) reduced auto-activation to a level that enabled successful screening (Supplementary Figure S1). XLG2 and XLG3 did not auto-activate.

Three Arabidopsis cDNA libraries designated GLUC, ROOT, and SAL (Klopffleisch et al., 2011) were screened using XLG baits. Briefly, the GLUC library was created from glucose treated, 7-day old *Arabidopsis thaliana* Col 0 seedlings grown in liquid culture (1% sucrose 7 days, no sucrose 2 days, 6% glucose 3 h), the ROOT library was created from 7-day-old roots, and the SAL library was created from *A. thaliana* suspension culture cells. The XLG2 bait was used only to screen the GLUC library. The primary candidate set (Supplementary Table S4) was culled of known Y2H artifacts (Venkatesan et al., 2009; Klopffleisch et al., 2011) and redundant entries to make a collection of 72 proteins of which 22 were found using XLG1, 3 were found using XLG2, and 49 were found using XLG3 baits. The full set of candidate XLG interacting proteins is provided in Table 1 with the corresponding confidence levels given by *P*-values. No single protein family was over represented and the set contained over 10% hypothetical proteins. We did not find the previously-reported XLG2 interactor nuclear protein RTV1 (Heo et al., 2012). While the previously-published PUB2 and 4 E3 ligases (Wang et al., 2017) where not found in this screen, the E3 ligase XBAT32 was found to interact with XLG1. XBAT32 is a RING-subtype whereas PUB2 and 4 are in the HECT subfamily of E3 ligases. XBAT2 is also found in the G protein interactome (Klopffleisch et al., 2011).

**Table 1:** Newly-discovered candidate XLG interacting proteins.

**Bait**	**Prey**	**Description**	**^a^Co-expression correlation coefficient**	**^b^p-value**	**^C^interaction**	**Source**
XLG1	At1g44170^*^	Aldehyde dehydrogenase 3H1	0.082	7.0E-17	Y2H/BiFC	ROOT
XLG1	At1g55450	S-adenosyl-L-methionine-dependent methyltransferases superfamily protein	−0.016	5.4E-03	Y2H	SAL
XLG1	At1g75240	Homeobox protein 33	0.074	1.3E-32	Y2H	GLUC
XLG1	At2g21160	Translocon-associated protein subunit alpha	0.076	2.3E-23	Y2H	SAL
XLG1	At2g21620	Dessication responsive protein	−0.010	3.1E-01	Y2H	Root
XLG1	At2g30160	Mitochondrial substrate carrier family protein	0.051	2.4E-02	Y2H	GLUC
XLG1	At2g34930	Disease resistance-like protein/LRR domain-containing protein	−0.042	2.2E-04	Y2H	ROOT
XLG1	At2g44450	Beta glucosidase 15	−0.022	1.3E-04	Y2H	SAL
XLG1	At3g04500	RNA recognition motif-containing protein	0.216	1.6E-23	Y2H	GLUC
XLG1	At3g11630	2-Cys peroxiredoxin BAS1	0.078	5.5E-22	Y2H	GLUC
XLG1	At3g55980^*^	Salt-inducible zinc finger 1	0.020	9.2E-04	Y2H/BiFC	ROOT
XLG1	At4g02380^*^	Senescence-associated protein SAG21	−0.005	2.5E-01	Y2H/BiFC	GLUC
XLG1	At4g09580^*^	SNARE associated golgi protein family	0.268	5.3E-31	Y2H	ROOT
XLG1	At4g18140	SCP1-like small phosphatase 4b	0.129	5.0E-33	Y2H	ROOT
XLG1	At4g19710	Bifunctional aspartokinase/homoserine dehydrogenase 2	0.120	3.9E-22	Y2H	GLUC
XLG1	At4g38770	Proline-rich protein 4	0.028	7.2E-09	Y2H	GLUC
XLG1	At4g39870	TLD-domain containing nucleolar protein	0.149	3.9E-15	Y2H	ROOT
XLG1	At5g06350	Rix1 complex component domain-containing protein	0.124	2.8E-17	Y2H	GLUC
XLG1	At5g42850	Thioredoxin-like protein Clot	0.024	1.1E-01	Y2H	SAL
XLG1	At5g54760	Translation initiation factor SUI1 family protein	0.106	9.7E-14	Y2H	SAL
XLG1	At5g57740	E3 ubiquitin-protein ligase XBAT32	−0.013	4.0E-01	Y2H	SAL
XLG1	At5g59880	Actin depolymerizing factor 3	0.054	3.7E-07	Y2H	SAL
XLG2	At4g40040	Histone H3.2	0.342	7.1E-33	Y2H	SAL
XLG2	At5g16470	Hypothetical protein	0.300	1.4E-27	Y2H	SAL
XLG2	At5g42050^*^	DCD (Development and Cell Death) domain protein	0.624	0.0E+00	Y2H/BiFC	GLUC
XLG3	At1g04040	HAD superfamily, subfamily IIIB acid phosphatase	−0.085	2.6E-21	Y2H	SAL
XLG3	At1g26380	FAD-binding and BBE domain-containing protein	0.099	3.2E-26	Y2H	GLUC
XLG3	At1g29930	Chlorophyll A/B binding protein 1	−0.058	5.3E-24	Y2H	GLUC
XLG3	At1g31780	Hypothetical protein	0.251	1.3E-17	Y2H	GLUC
XLG3	At1g54780	Hypothetical protein	−0.077	1.3E-27	Y2H	GLUC
XLG3	At1g67320	Probable DNA primase large subunit	0.169	1.5E-19	Y2H	GLUC
XLG3	At1g70200	RNA recognition motif-containing protein	−0.062	4.8E-08	Y2H	GLUC
XLG3	At1g70770	Hypothetical protein	0.378	6.4E-76	Y2H	GLUC
XLG3	At1g71410^*^	Putative protein kinase	0.318	9.3E-34	Y2H/BiFC	ROOT
XLG3	At1g73030^*^	ESCRT-related protein CHMP1A	0.186	2.7E-13	Y2H/BiFC	ROOT
XLG3	At1g76160	SKU5-like 5 protein	0.011	3.3E-01	Y2H	SAL
XLG3	At2g01140	Fructose-bisphosphate aldolase 3	0.219	6.4E-47	Y2H	SAL
XLg3	At2g04410	RPM1-interacting protein 4-like protein	0.143	4.8E-10	Y2H	GLUC
XLG3	At2g21170	Triosephosphate isomerase	0.245	1.3E-34	Y2H	GLUC
XLG3	At2g25970	Hypothetical protein	0.567	6.8E-148	Y2H	GLUC
XLG3	At2g27900	Hypothetical protein	0.538	2.0E-105	Y2H	GLUC
XLG3	At2g30490	Trans-cinnamate 4-monooxygenase	0.148	1.1E-34	Y2H	SAL
XLG3	At2g30860	Glutathione S-transferase PHI 9	0.089	6.0E-11	Y2H	GLUC
XLG3	At2g30960	Hypothetical protein	0.339	5.6E-91	Y2H	SAL
XLG3	At2g33040	ATP synthase subunit gamma	0.235	5.8E-28	Y2H	SAL
XLG3	At2g34410	O-acetyltransferase-like protein			Y2H	GLUC
XLG3	At2g38480^*^	CASP-like protein	−0.135	1.3E-12	Y2H/BiFC	GLUC
XLG3	At2g40140^*^	Zinc finger CCCH domain-containing protein 29	0.137	1.1E-35	Y2H/BiFC	GLUC
XLG3	At2g41430^*^	Dehydration-induced protein ERD15	0.162	2.0E-32	Y2H	GLUC
XLG3	At2g43620	Chitinase family protein	0.025	3.1E-04	Y2H	GLUC
XLG3	At3g02120	Hydroxyproline-rich glycoprotein-like protein	0.040	8.2E-04	Y2H	SAL
XLG3	At3g03780	Methionine synthase 2	0.045	4.8E-04	Y2H	GLUC
XLG3	At3g16420	PYK10-binding protein 1	0.005	3.7E-01	Y2H	GLUC
XLG3	At3g19640^*^	Magnesium transporter MRS2-3	0.516	1.2E-135	Y2H/BiFC	GLUC
XLG3	At3g19820	Delta(24)-sterol reductase	0.119	1.7E-26	Y2H	GLUC
XLG3	At3g26520^*^	Aquaporin TIP1-2	−0.068	9.5E-16	Y2H	ROOT
XLG3	At3g27090^*^	DCD (Development and Cell Death) domain protein	0.168	1.4E-11	Y2H/BiFC	SAL
XLG3	At3g42050^*^	V-type proton ATPase subunit H	0.267	2.1E-21	Y2H	GLUC
XLG3	At3g44100	Hypothetical protein	0.274	1.1E-31	Y2H	SAL
XLG3	At3g60210^*^	GroES-like family protein	−0.049	3.8E-02	Y2H	ROOT
XLG3	At3g60750	Transketolase	−0.050	6.2E-05	Y2H	GLUC
XLG3	At4g14520	DNA-directed RNA polymerase II-like protein			Y2H	GLUC
XLG3	At4g15910^*^	DROUGHt-INDUCED 21	0.128	4.7E-70	Y2H	SAL
XLG3	At4g28610^*^	Phosphate starvation response 1 protein	0.483	1.5E-83	Y2H	GLUC
XLG3	At4g37180	myb family transcription factor	0.273	2.3E-62	Y2H	GLUC
XLG3	At5g06310	protection of telomeres 1b			Y2H	GLUC
XLG3	At5g17670	Hydrolase-like protein	−0.126	1.2E-23	Y2H	GLUC
XLG3	At5g17920	5-methyltetrahydropteroyltriglutamate–homocysteine methyltransferase			Y2H	GLUC
XLG3	At5g28740	tetratricopeptide repeat domain-containing protein	0.539	1.1E-145	Y2H	SAL
XLG3	At5g44340^*^	Tubulin beta chain 4	0.046	1.8E-02	Y2H	GLUC
XLG3	At5g45760	Transducin/WD40 domain-containing protein	0.402	6.9E-71	Y2H	GLUC
XLG3	At5g66240^*^	Transducin family protein / WD-40 repeat family protein	0.516	1.0E-97	Y2H/BiFC	GLUC

The co-expression correlation coefficient indicated the regression linear results based on 1779 arrays. The

a co-expression correlation ranged from −1 to +1, represented the correlation direction, closer to +1, the more positive relation. The

b *p-value indicated the strength of the correlation*.

C In the interaction section, the Y2H means the preys were obtained from the Y2H screening from the corresponding baits, and the BiFC indicated this confirmation was performed on both XLG1 and XLG3 full length. The prey proteins used for the BiFC assay marked with ^*^.

### GO analysis indicates enrichment in stress response

Urano et al. (2016) quantitated 30 phenotypic traits of the *xlg1,2,3* triple mutant, including all the known *agb1* mutant phenotypes, and concluded that XLG proteins are important for many physiologies and development. The single mutant alleles of the three *XLG* genes have wild type hypocotyl lengths and primary root lengths (Ding et al., 2008), and showed no differences in response to salt, tunicamycin and D-glucose in post-germination development compared to wildtype seedlings (Chakravorty et al., 2015). Only the single mutants of *XLG2* have altered plant immunity responses (Maruta et al., 2015). Therefore, we analyzed for predicted functions of the XLG interactors.

**Figure 1:**
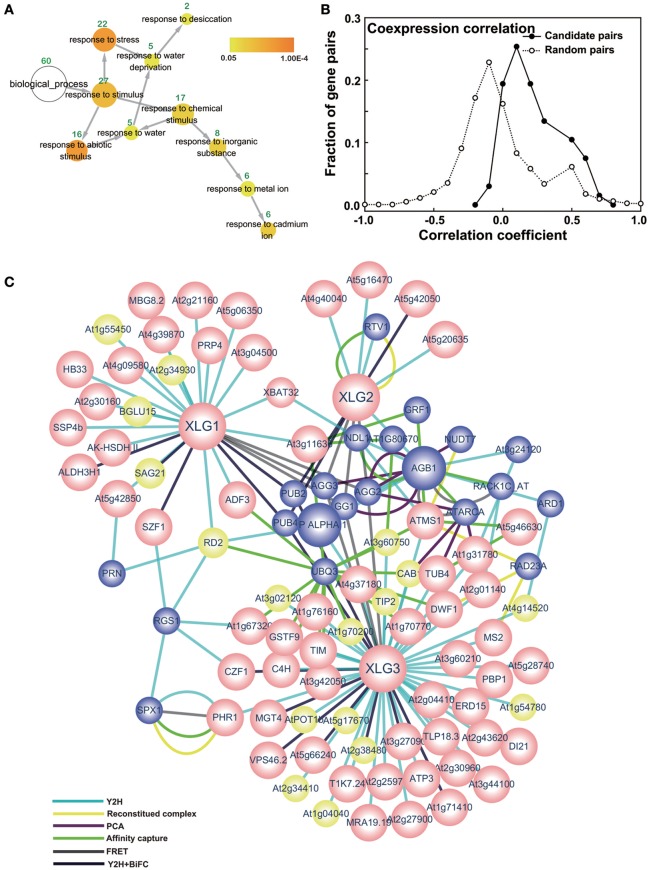
Core XLG interactome. **(A)** GO enrichment of the interactors. The GO analysis was performed in cytoscape using all the interactors from the Y2H screen. The yellow nodes are significantly represented (*p* < 0.05) and the white node represents the entire set. The color bar indicates the correction *p*-value for each categories, which was done by Benjamini & Hochberg False Discovery Rate (FDR) correction. The area of a node is proportional to the number of genes (green numbers labeled above the nodes) in the test set annotated to the corresponding GO category. Arrows indicate the hierarchy of the categories. **(B)** Co-expression coefficient distribution of candidate and random pairs. The co-expression coefficient between the 67 pairs of baits and prey were calculated using the online software CressExpress (http://cressexpress.org/). The solid line indicates candidate protein pairs discovered here and the dotted line indicates random protein pairs. Student *t*-test was performed in SAS8.0 and the *p* = 2E-4. **(C)** Core XLG interactome network. The 72 interactors from the Y2H screen plus additional candidates that have at least 2 edges are shown. The nodes with pink and yellow color indicate interactors identified here and blue nodes are taken from the BioGRID database and the literature (Wang et al., 2017). Pink nodes designate an interactor that has a positive co-expression coefficient, and yellow nodes have a negative co-efficient. Different edge colors represent the different interaction methods as indicated.

Gene Ontogeny (GO) analysis was performed for the candidate set of interactors using BiNGO in cytoscape (Maere et al., 2005). For the “biological_process” analysis, the cutoff *p*-value was set to 0.05. As shown in Figure 1A, the main GO enrichment for all the interactors were stress and abiotic stimuli. These results were consistent with the G protein interactors enriched in biotic/abiotic stresses, developmental processes and cell organization, and biogenesis (Klopffleisch et al., 2011). GO analysis of the current set of interactors also revealed proteins involved in amino acids biosynthesis and metabolic process, including methionine, homocysteine, sulfur amino acid and aspartate family amino acid (Supplementary Table S1).

### Correlation of gene expression supports the reliability of the XLG interactome

Correlated expression is a Predictor of co-functionality of genes in common pathways and processes (Bhardwaj and Lu, 2009). Protein partners, in order to functionally interact, must be expressed in the same cells, compartments, and induced or repressed by the same conditions. Therefore, we analyzed co-expression of corresponding genes between the 72 pairs of interactors and corresponding *XLG* genes using the online software CressExpress (http://cressexpress.org/). CressExpress performs linear regression using expression values harvested from publicly-available microarray data. We used version 3 containing 1779 arrays and included all the experimental results in our analyses. After the analyses, Pearson correlation coefficients and *p*-values were generated to evaluate the co-expression relationship between the two genes. The co-expression correlation coefficient ranged from −1 to +1, with +1 indicating a perfect positive correlation and the *p*-value indicated the strength of the co-expression (Wei et al., 2006). The co-expression relationship between 4 of the 72 pairs was not found. The results showed 62 out of 68 (91.17%) display significantly positive or negative correlation coefficient and 52 of them (76.47%) had positive correlation. Figure 1B shows the distribution of the correlation coefficient of the 68 protein pairs from the interactome and 2000 random selected pairs. The randomly-selected gene pairs formed a normal distribution around a correlation coefficient of −0.1. In contrast, the distribution of the candidate gene pairs was bimodal and right shifted with the maxima at 0.1 and 0.5). A Student *T*-test showed that the distribution of candidate gene pairs was significantly right-shifted from random pairs (*p* = 2E-4) indicating that the co-expression of the gene pairs is not random.

### XLG partners direct subcellular localization of XLG proteins

**Figure 2:**
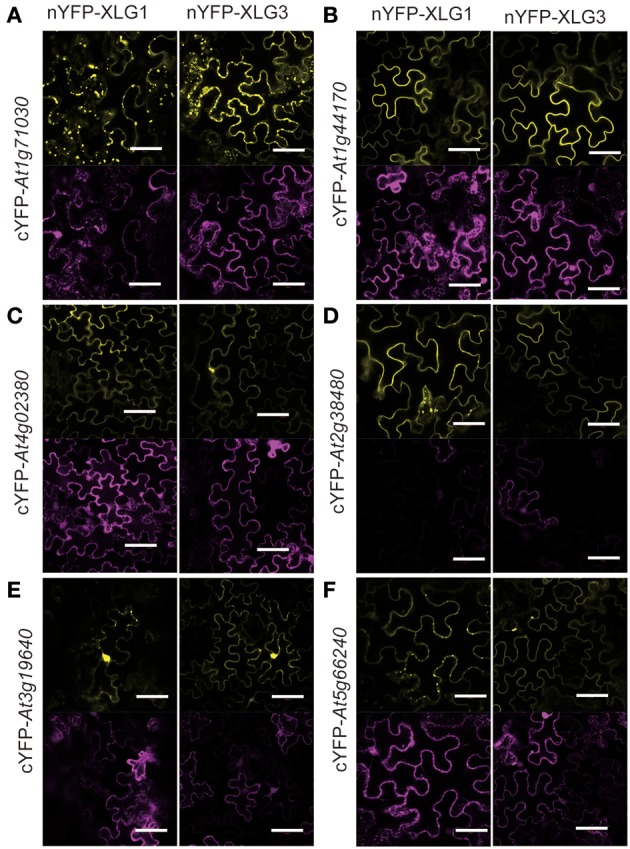
BiFC for selected candidate XLG1 and 3 interacting proteins outside the nucleus. **(A)** At1g71030 **(B)** At1g44170 **(C)** At4g02380 **(D)** At2g38480 **(E)** At3g19640 **(F)** At5g66240. The XLGs were fused to the N-terminus of YFP and the prey proteins were fused to the C-terminus of YFP. The bait constructs nYFP-XLG1 and XLG3 are indicated at the top columns and the prey constructs are listed at the left rows. The transformation control is the mitochondria marker MT-RK. Each combination of the prey and bait has two panels; the upper one is the YFP signal indicated by complementation of cYFP and nYFP and the lower one is RFP indicating positive transformation. Bar = 50 μm

**Figure 3:**
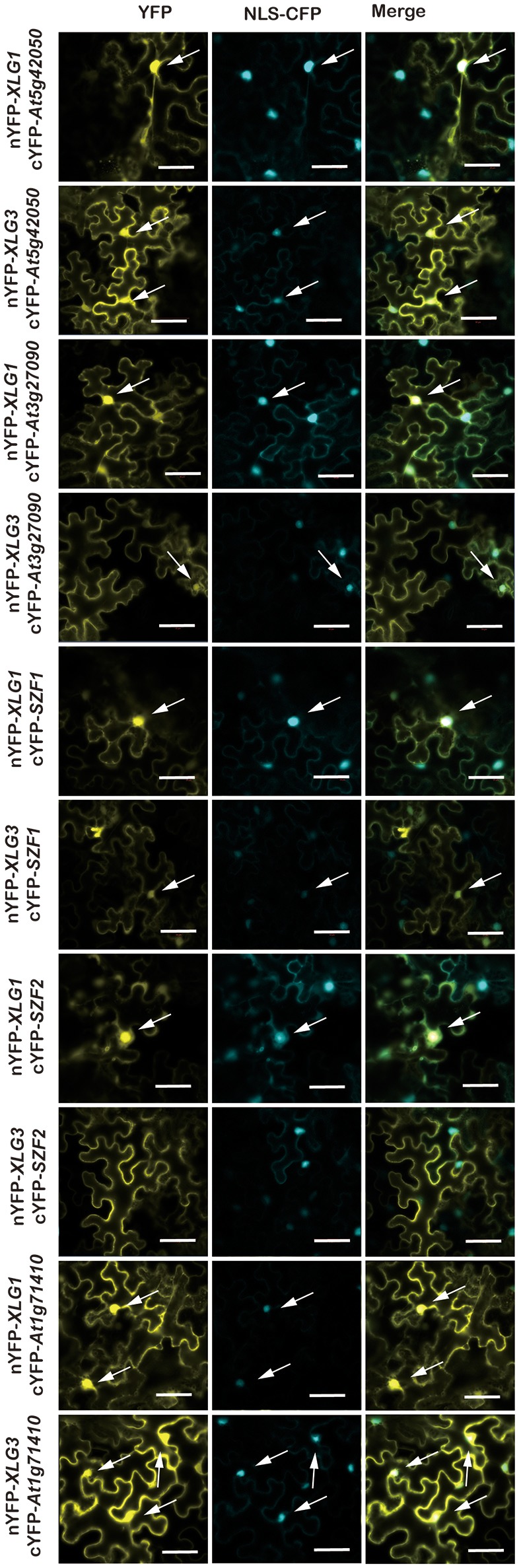
BiFC for selected candidate XLG interacting proteins in the nucleus. The indicated XLG proteins fused to the N-terminus of YFP with the prey proteins fused to the C-terminus of YFP is provided on the left rows. Positive transformation was confirmed by the nucleus marker NLS-CFP. Each combination of pairs shows three panels: the first column is the YFP signal indicated by fluorescence complementation of cYFP and nYFP, the second column is the CFP fluorescence of the nucleus marker, NLS-CFP (Nucleus Localization Signal CFP), and the last column is the merged images of the first two columns. Bar = 50 μm. The white arrows indicate a Spearman coefficient that is 0.75 or greater.

To ascertain the quality of the set of potential XLG interactors, 18 candidates were tested for XLG interaction *in vivo* using Bimolecular Fluorescence Complementation (BiFC) (Table 1, marked with^*^). Eleven (>60%) were confirmed by BiFC to interact with both XLG1 and XLG3 bait *in vivo* (Figures 2, 3). Supplementary Figure S2 provides the negative controls used in these assays. The interaction partner affects the localization. For example, *At1g73030*, which encodes an ESCRT-related protein that co-localizes to the plasma membrane and endosome (Tian et al., 2007; Spitzer et al., 2015) interacted with XLG1 and XLG3 in punctate structures (Figure 2A). *At1g44170*, encodes an aldehyde dehydrogenase induced by ABA and dehydration, and interacts with XLG1 and XLG3 on the plasma membrane (Figure 2B). SZF1 and SZF2 are two transcription factors that interact with XLG1 and XLG3 in the nucleus (Figure 3). This partner-dependent localization explains the inconsistencies in the literature (Ding et al., 2008; Heo et al., 2012; Chakravorty et al., 2015; Maruta et al., 2015; Wang et al., 2017).

### Core XLG interactome

An interactome network (Figure 1C) was constructed based on the interactors identified herein together with potential interactions assembled from the BioGRID interactome database (http://thebiogrid.org). Nodes were included based on the following rules. Yellow and pink nodes are proteins identified in the present study that interact with the indicated XLG protein noted by the corresponding edges. Yellow nodes are XLG-interacting proteins that are negatively correlated whereas pink nodes are positively correlated based on the co-expression analyses. Blue nodes are proteins from public data that have at least two edges.

### XLGs interact with SZFs and DCDs in the nucleus

As discussed above, some of the XLG protein-interactor pairs appeared to be nuclear localized. To determine the confidence level of the nuclear localization, we included the nuclear marker NLS-CFP and calculated the Spearman Rank Order coefficient (French et al., 2008) which measures the strength and direction of association between the nucleus marker NLS-CFP and the XLG interactors. As shown in Supplementary Figure S3, the box plot showed that the percentile line is above 0.2 and the median values for most is above 0.5 (exception is the XLG3 and *SZF2* pair), indicating a strong positive correlation and therefore high confidence that the XLG interaction with the indicated partners mainly occurs in the nucleus.

**Figure 4:**
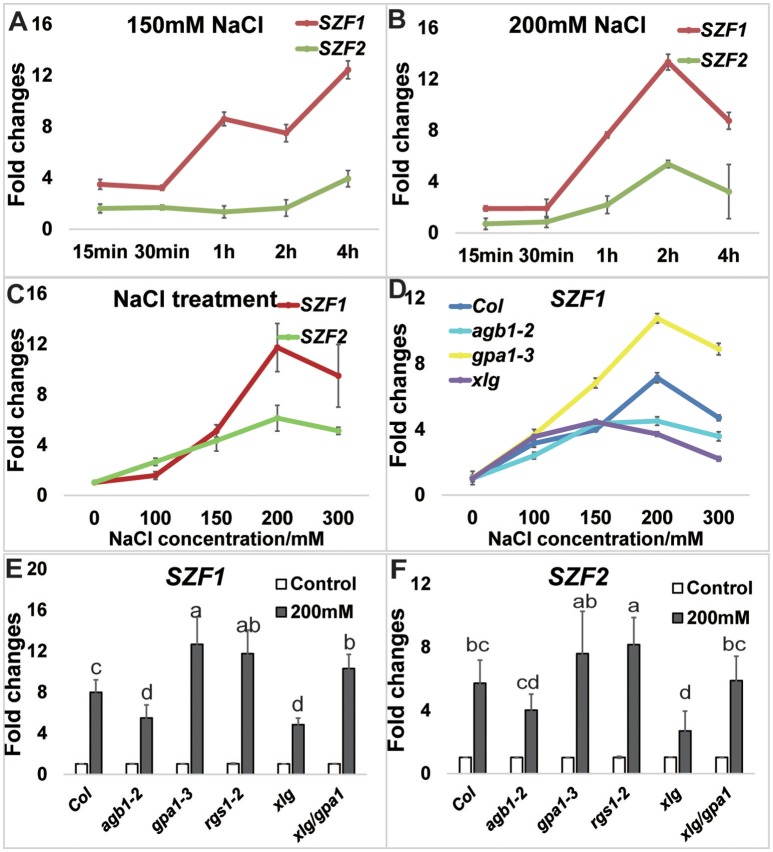
NaCl-induced expression of *SZFs*. Seven-day-old *Arabidopsis* seedlings grown hydroponically in dim light room were used for the treatment. **(A)** The expression level of *SZF1* and *SZF2* in the time course of 0, 15, 30 min, 1, 2, and 4 h in the 150 mM treatment as described in the Materials and Methods. **(B)** The expression level of *SZF1* and *SZF2* in the time course of 0 min–4 h after the 200 mM treatment. **(C)** The expression level of *SZF1* and *SZF2* in the NaCl dose responses of 0, 100, 150, 200 mM. **(D)** The expression level of *SZF1* and *SZF2* in the NaCl dose responses of 0, 100, 150, 200 mM in the mutants of *Col, agb1-2, gpa1-3, xlg1/2/3*. **(E,F)** The expression level of SZF1 **(E)** and SZF2 **(F)** in response to 200 mM NaCl for 2 h between Col and G protein mutants [*agb1-2, gpa1-3, rgs1-2, xlg1/xlg2/xlg3* triple (indicated *xlg*) and *xlg1/xlg2/xlg3/gpa1-3* quadruple (indicated *xlg/gpa1*)]. ANOVA analysis was performed using SAS8.0 set at a *p* = 0.05 with five biological replicates.

DCD domain protein, also called N-rich protein (NRP) (Ludwig and Tenhaken, 2001), is induced during the hypersensitive reaction caused by microbial pathogen and involved in development and death (Tenhaken et al., 2005). *At5g42050*, a DCD domain protein was reported to show signal translocated from cytosol to mitochondria during stress treatment (Hoepflinger et al., 2011). We did not observe this; our studies showed that *At5g42050* is localized in the cytoplasm and nucleus (Supplementary Figure S3), which is consistent with our BiFC results (Figure 4). Moreover, subcellular localization of *AtSZF1* and *AtSZF2* in the protoplast was cytoplasmic and nuclear (Supplementary Figure S4), in contrast to published data that showed SZF1 is only nuclear localized in onion epidermal cells (Sun et al., 2007).

### *SZF1* and *2* Are induced by salt in an XLG-dependent manner

Previous reports showed that the expression of *SZF1* and *2* is transiently induced by NaCl (Sun et al., 2007) but the concentration of NaCl they used was not provided and it is not known if the conditions they used extrapolate to conditions tested here. Therefore, we determined the kinetics and dose-dependency for *SZF1* and *2* gene expression induced by NaCl under our lab conditions.

**Figure 5:**
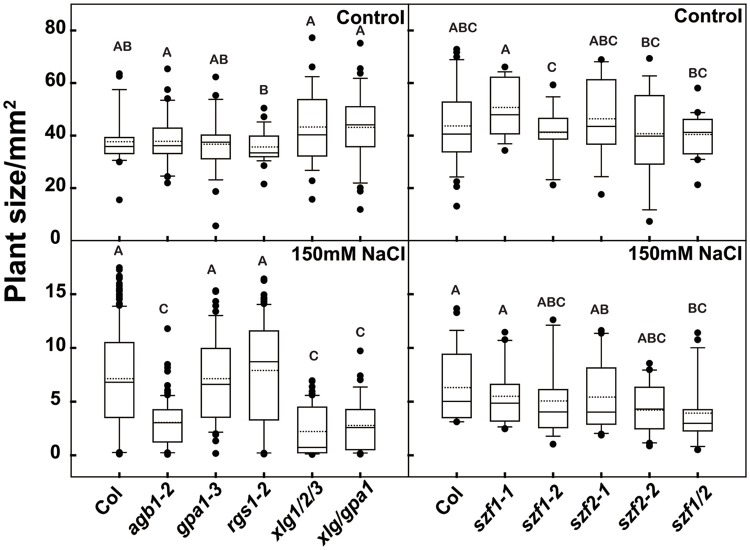
Salt phenotypes of the G protein and *szf* mutants. Arabidopsis seedlings were grown horizontally on ¼ MS medium with 1% sucrose with or without 150 mM NaCl under continuous dim light for 2 weeks, and the leaf area were measured to evaluate the growth status of the plants. The box plot indicates the distribution leaf area. The solid line in the box indicated the median and the dot line indicated the mean value. The bottom and the top of the box represented first and the third quartiles. The start and the end of the whiskers represented the maximum and minimum of the value. The dots represented the outliers. Different lowercases letter indicate significant differences (*p* < 0.05) between any two genotypes. The ANOVA analysis was performed using SAS8.0, *n* ≥ 24.

Seven-day-old Arabidopsis seedlings were grown under dim light (60–70 μEm^−2^s^−1^) for the gene expression analyses. Seedlings were treated with either 150 mM or 200 mM NaCl and sampled at 0, 15 min, 30 min, 1, 2, and 4 h. Treatment with 150 mM NaCl increased expression of *SZF1* and *SZF2* over 4 h (Figure 4A); with 200 mM NaCl treatment (Figure 4B), the expression level of *SZF1* and *SZF2* peaked at 2 h. Note that this peak time is different than that published by Sun et al. (2007). A dose response was determined using 2 h as the endpoint. The expression level of *SZF1* and *SZF2* peaked at 200 mM NaCl (Figure 4C). Having established the optimal NaCl dose for SZF gene expression and the timing of the peak expression under the conditions used here, we next determined if *SZF* gene expression is altered in the G protein mutants. The *SZF* gene expression level of the G protein mutants, *agb1-2, rgs1-2, gpa1-3, xlg1/2/3* triple, and *xlg1/2/3/gpa1-3* quadruple was tested at 200 mM NaCl for 2 h (with 5 biological replicates). *SZF1* gene expression in the wild type increased 7-fold whereas *SZF1*gene expression in the *agb1-2* and *xlg1/2/3* triple mutants showed a lower response to the NaCl treatment (Figure 4E). The *gpa1-3* single, *xlg1/2/3/gpa1-3* triple, and *rgs1-2* single mutants showed higher expression level in response to NaCl treatment. For *SZF2*, the gene expression level of the wild type increased 5-fold, and just as for *SZF1* expression, the expression level is lower in the *agb1-2* single and *xlg1/2/3* triple mutants and higher in the *gpa1-3* and *rgs1-2* mutants (Figure 4F). We also tested the expression of genes encoding the salt induced DCD domain proteins *At5g42050* (Hoepflinger et al., 2011), and found no differences between the G protein mutants and the wild type (Supplementary Figure S5) suggesting that the reduction of the NaCl-induced *SZF* gene expression in the *xlg* triple mutant is specific. Finally, salt resistance of the G protein and SZF mutants were tested. The *agb1*-2 single, *xlg* triple and *xlg1/xlg2/xlg3/gpa1-3* quadruple mutants were hypersensitive to salt stress under 150 mM NaCl treatment (Figure 5), consistent with previous reports (Colaneri et al., 2014; Yu and Assmann, 2015; Urano et al., 2016). The single mutants of *szf1* and *szf2* had wild type sensitivity to NaCl whereas the double szf1/2 mutants were hypersensitive (Figure 5) in agreement with previous findings (Sun et al., 2007). A NaCl sensitivity phenotype for the *szf1/2* double mutants is consistent with our observation that gene expression of *SZF1/2* is salt-inducible (Figure 4, Supplementary Table S3). Note that the reduced expression of *SZF1/2* in the *xlg1/2/3/gpa1* quadruple mutants was rescued to wildtype level by loss of the *gpa1*-3 null mutant (Figures 4E,F), whereas in the salt stress experiment, the growth of *xlg1/2/3/gpa1* was not fully rescued by loss of GPA1, although it showed slight recovery. This is because at 150–200 mM NaCl, the response is saturated. When retested at 75 mM NaCl, loss of GPA1 fully rescued the salt hypersensitivity of *xlg1/2/3* triple mutant (Supplementary Figure S6).

**Figure 6:**
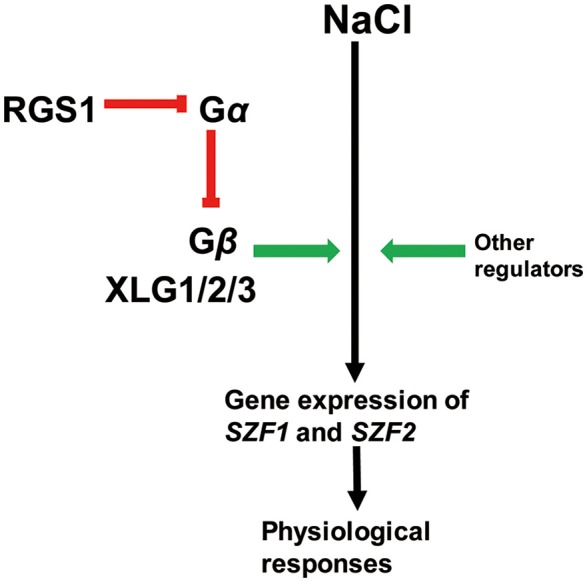
A putative model for the G protein regulated salt stress responses. In this model, RGS1 inhibit the function of Gα from releasing the Gβ subunits. The Gβ subunit interact with XLG1/2/3 to enhance the plants growth through expression of *SZF1* and *SZF2*. Green arrows indicate positive regulation and the red one indicates inhibition. The black arrow represents processes in NaCl responsiveness.

According to the salt stress phenotype data and the gene expression data, we propose a working model of the role of G protein signaling during salt stress (Figure 6). In this model, the RGS1 protein interacts with Gα to inhibit the Gα from releasing Gβ subunits, and Gβ subunits interact with XLGs to regulate the gene expression of *SZF1* and *SZF2*. The expression of *SZF1* and *SZF2* enhances the growth of plants under saline stress. We include a modulatory role for other regulators based on the literature.

### Newly-identified partners for XLG-mediated responses

Among the 72 candidates, 5 are “in response to water deprivation,” namely *At2g41430 (ERD15), At4g15910 (DI21), At4g02380 (SAG21), At2g21620(RD2)*, and *At1g44170 (ALDH3H1)* (Supplementary Table S1). *ERD15* (Early Response to Drought) is an attenuator of ABA responses and regulates stomatal aperture (Aalto et al., 2012). Overexpression of *ERD15* sensitizes plants to drought stress (Kariola et al., 2006). Plant aldehyde dehydrogenases (ALDHs) contains 13 distinct families encoding aldehyde dehydrogenases which catalyze the oxidation of reactive aldehydes to their corresponding carboxylic acids using NAD (P)^+^ as a cofactor (Kirch et al., 2001; Stiti et al., 2011; Brocker et al., 2013). ABA, NaCl, and drought increases the expression level of the aldehyde dehydrogenase 3H1 gene (*ALDH3H1*) in the root (Missihoun et al., 2012). *ALDH3H1* gene expression is important for long-term adaptation. G proteins regulate the drought stress response through multiple strategies. GPA1 regulates transpiration efficiency and stomatal density (Zhang et al., 2008; Nilson and Assmann, 2010). AGB1 is important for drought tolerance (Xu et al., 2015). AtRGS1 plays a role in ABA and drought tolerance (Chen et al., 2006). Phospholipase D (PLD) is involved in the osmotic stress response through hydrolysis of phosphatidic acid (PA), the ABA signaling pathway (Jacob et al., 1999) and the biosynthesis of proline (Thiery et al., 2004). PDLα 1 interacts with GPA1 (Thiery et al., 2004) and AtRGS1 (Choudhury and Pandey, 2016), and the product of PLDα1, phosphatidic acid, may slightly inhibit the GAP activity of AtRGS1 (Choudhury and Pandey, 2017).

The data suggest that XLG is involved in trafficking. The interactors ESCRT (endosomal sorting complexes required for transport)-related CHARGED MULTIVESICULAR BODY PROTEIN/CHROMATIN MODIFYING PROTEIN1A (CHMP1A; *At1g73030*) and CHMP1B (*At1g17730*) proteins are essential for embryo and seedling development (Spitzer et al., 2009).

As shown in Figure 1C, PHR1 interacts with SPX1, which itself interacts with AtRGS1. SPX1 is an inhibitor of PHR1 (Puga et al., 2014). The *phr1* (At4g28610) mutant is defective in the Pi starvation response (Rubio et al., 2001) and cooperates with another protein PHL1 (At5g29000) to regulate root hair development in response to phosphate starvation (Bustos et al., 2010). This information suggests that G proteins play a part in phosphate sensing and regulation.

Annotation of “Cadmium Stress” ranks highest in the G protein interactome (Klopffleisch et al., 2011). In the present study on XLG interactors, cadmium stress also appeared. There are four genes annotated as response to cadmium, namely *At2g01140, At3g03780 (MS2), At5g17920 (ATMS1), At3g60750* (Supplementary Table S1). A null mutation in the rice *OsDEP1*, encoding the gamma subunit of the G protein, confers cadmium stress on yeast cells and plants (Kunihiro et al., 2013). The *xlg1/2/3* triple mutants and *agb1*-2 are hypersensitive to cadmium stress (Urano et al., 2016).

### Summary

The number of studies on XLGs grew slowly since the first plant extra-large G protein was reported (Lee and Assmann, 1999), however, in the last 2 years, interest in these atypical signal components has surged relatively (Chakravorty et al., 2015; Maruta et al., 2015; Liang et al., 2016; Urano et al., 2016; Wang et al., 2017). The general role for XLG proteins centers on stress responsiveness but we still lack a good understanding of the mechanism and while the subcellular localization has been reported, there has been conflicting results with no explanation. Our work provides a large set of stress-related proteins for future studies to test mechanism. We also provide an explanation of the conflicting reports on XLG subcellular localization by showing that the localization of the XLGs is dependent on the specific interacting partner.

## Author contributions

YL designed experiments, collected the data, prepared the figures, and wrote the manuscript. AJ wrote the manuscript and YG and YL edited the manuscript. AJ managed the project. All authors read and approved the final manuscript.

### Conflict of interest statement

The authors declare that the research was conducted in the absence of any commercial or financial relationships that could be construed as a potential conflict of interest.

## Assurance

The interactors described here will be deposited into the Arabidopsis G protein interactome upon acceptance for publication. Website: http://bioinfolab.unl.edu/emlab/Gsignal/index.pl

## Supplementary material

Supplementary Figure S1 Autoactivation test for the *XLG1, XLG2* and *XLG*3 in yeast. Yeast AH109 transformed with XLG1-pAS2-1, XLG2-pAS2-1 and XLG3-pAS2-1 were grown on dropout (DO) medium –Trp (W) to confirm transformation and cell viability. Autoactivation was assayed on DO-Trp-His-Ade supplemented with the indicated concentrations of 3-AT (0-20mM).

Supplementary Figure S2 Negative controls for the BiFC. In all assays, positive transformation was confirmed by the mitochondria marker MT-RK. The combination of bait constructs nYFP-XLG1 and XLG3 and the prey constructs were listed above the figures. Each combination of the prey and bait had two panels, the upper one was the YFP signal indicated the complementary of the cYFP and nYFP and the lower one was RFP which was transformation control. Bar = 50 μm

Supplementary Figure S3 Spearman Rank Order coefficients for nuclear localization. This coefficient is to examine the colocalization between the nucleus marker NLS-CFP and the yellow fluorescence. The range of the coefficient is from −1, a strong negative correlation, to +1, a strong positive correlation. The *p*-value above 0.195 (the spearman coefficient when, *p* < 0.05, degree of freedom = 100) is positive correlated. Box plots indicate the distribution of the coefficient and the number of the samples labeled in the figure. The solid line inside the box indicates the median and the dotted line indicates the mean value. The bottom and the top of the box represent first and third quartiles. The start and the end of the whiskers represented the maximum and minimum of the values. The dots represent the outliers.

Supplementary Figure S4 Subcellular localization of *SZF1, SZF2* and DCD protein *At5g42050*. Protoplasts were isolated from 5-week-old *Arabidopsis Col* and transformed with GFP-*SZF1*, GFP-*SZF2* and GFP-*At5g42050* in addition to the nucleus marker Histone-2B-RFP. The constructs (GFP-*SZF1*, GFP-*SZF2* and GFP-*At5g42050*) are listed to the left of the images. Each construct has three panels from left to right, **A**, **D** and **G**, GFP signal are the expression patterns of theindicated target proteins; (**B**, **E**, and **H**), RFP signal is the nucleus signal; (**C**, **F**, and **I**), the merge images of GFP and RFP. Bar = 10 μm.

Supplementary Figure S5 Salt-induced DCD domain protein *At5g42050* gene expression. Seven-day-old *Arabidopsis* seedlings *Col* and G protein mutants (*agb1-2, gpa1-3, rgs1-2, xlg1/xlg2/xlg3* and *xlg1/xlg2/xlg3/gpa1-3*) grown hydroponically in dim light room were treated with 200mM NaCl for 2h. ANOVA analysis with SAS8.0, *p* < 0.05, five biological replicates.

Supplementary Figure S6 Salt phenotypes of the G protein under mild salt stress. Seedlings of the indicated genotypes were grown in 1/2 MS medium with or without 75mM NaCl for 18 days. Box plot indicates the distribution leaf areas. The solid line in the box indicates the median and the dot line indicates the mean value. The bottom and the top of the box represent first and third quartiles. The start and the end of the whiskers represent the maximum and minimum of the values. The dots represent the outliers. Different lowercases letter indicated significant differences (*p* < 0.05) between any two genotypes. The ANOVA analysis with SAS8.0, *n* = 24.

Supplementary Table S1 GO enrichment analysis of the interactors. This excel file provides the GO analysis results from the BiNGO. The *p* < 0.05, and the correction *p*-value was performed by Benjamini & Hochberg False Discovery Rate (FDR) correction.

Supplementary Table S2 The primers used in this paper. Shown are the primers used for the indicated TOPO cloning and the qRT-PCR reactions.

Supplementary Table S3 The quantitative fold changes for the real time PCR. Shown are the quantitative fold changes for the real time PCR in corresponding to Figures 4E,F and Figure S5. ANOVA analysis with SAS8.0, *p* < 0.05, five biological replicates. The value indicates the standard error.

Supplementary Table S4 The raw data for the Y2H. This table provides the raw data for the Y2H without filtering the published artifacts. The known artifacts were in red.
