# Supplementary figures and images for: Extra Large G-Protein Interactome Reveals Multiple Stress Response Function and Partner-Dependent XLG Subcellular Localization
Source: Front Plant Sci. 2017 Jun 13;8:1015. doi: 10.3389/fpls.2017.01015 (PMC5469152; doi:10.3389/fpls.2017.01015)

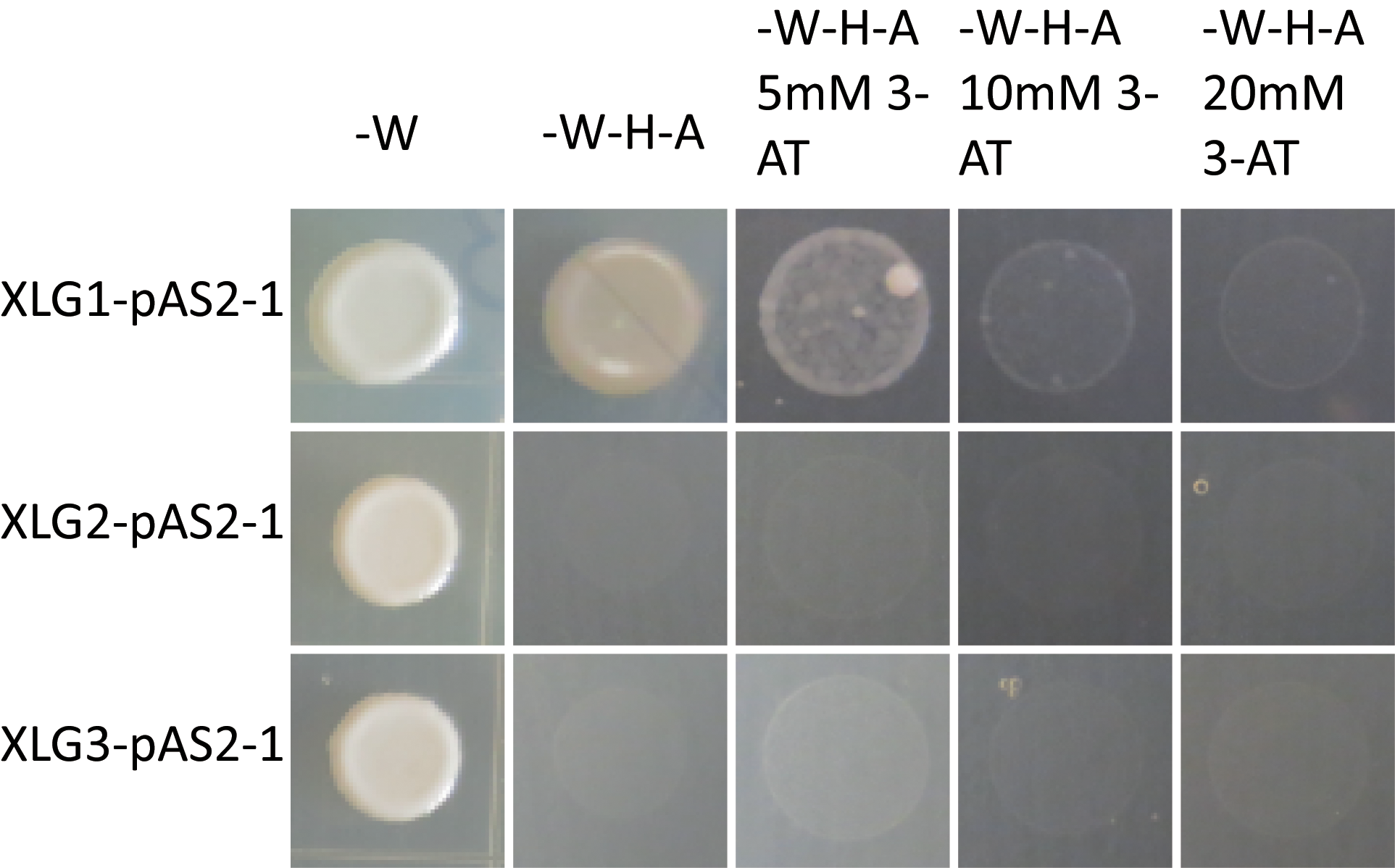

Supplement: Supplementary Figure S1 — Autoactivation test for the XLG1, XLG2 and XLG3 in yeast. Yeast AH109 transformed with XLG1-pAS2-1, XLG2-pAS2-1 and XLG3-pAS2-1 were grown on dropout (DO) medium –Trp (W) to confirm transformation and cell viability. Autoactivation was assayed on DO-Trp-His-Ade supplemented with the indicated concentrations of 3-AT (0-20mM). [file Image1.TIF]

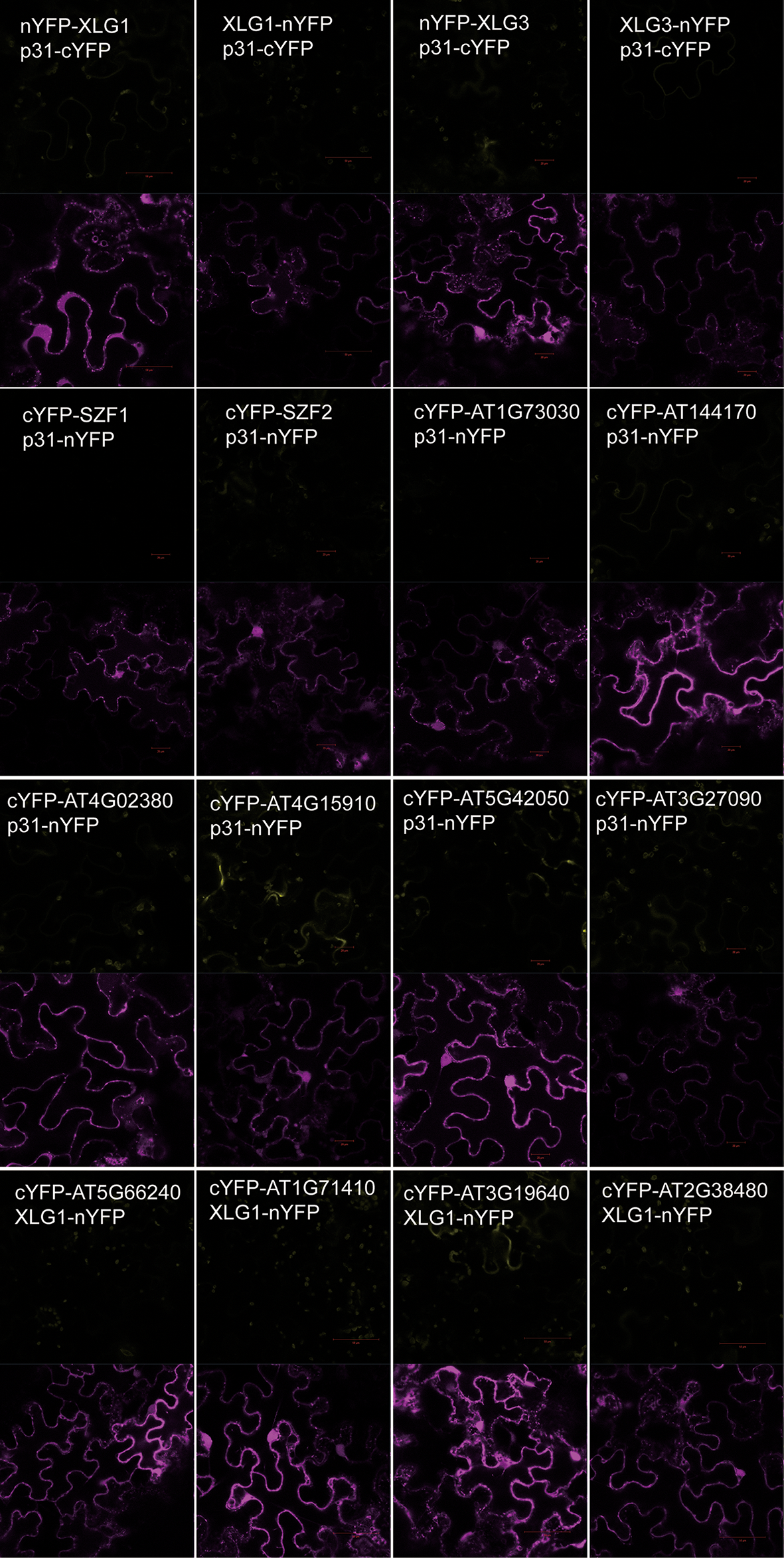

Supplement: Supplementary Figure S2 — Negative controls for the BiFC. In all assays, positive transformation was confirmed by the mitochondria marker MT-RK. The combination of bait constructs nYFP-XLG1 and XLG3 and the prey constructs were listed above the figures. Each combination of the prey and bait had two panels, the upper one was the YFP signal indicated the complementary of the cYFP and nYFP and the lower one was RFP which was transformation control. Bar = 50 μm [file Image2.TIF]

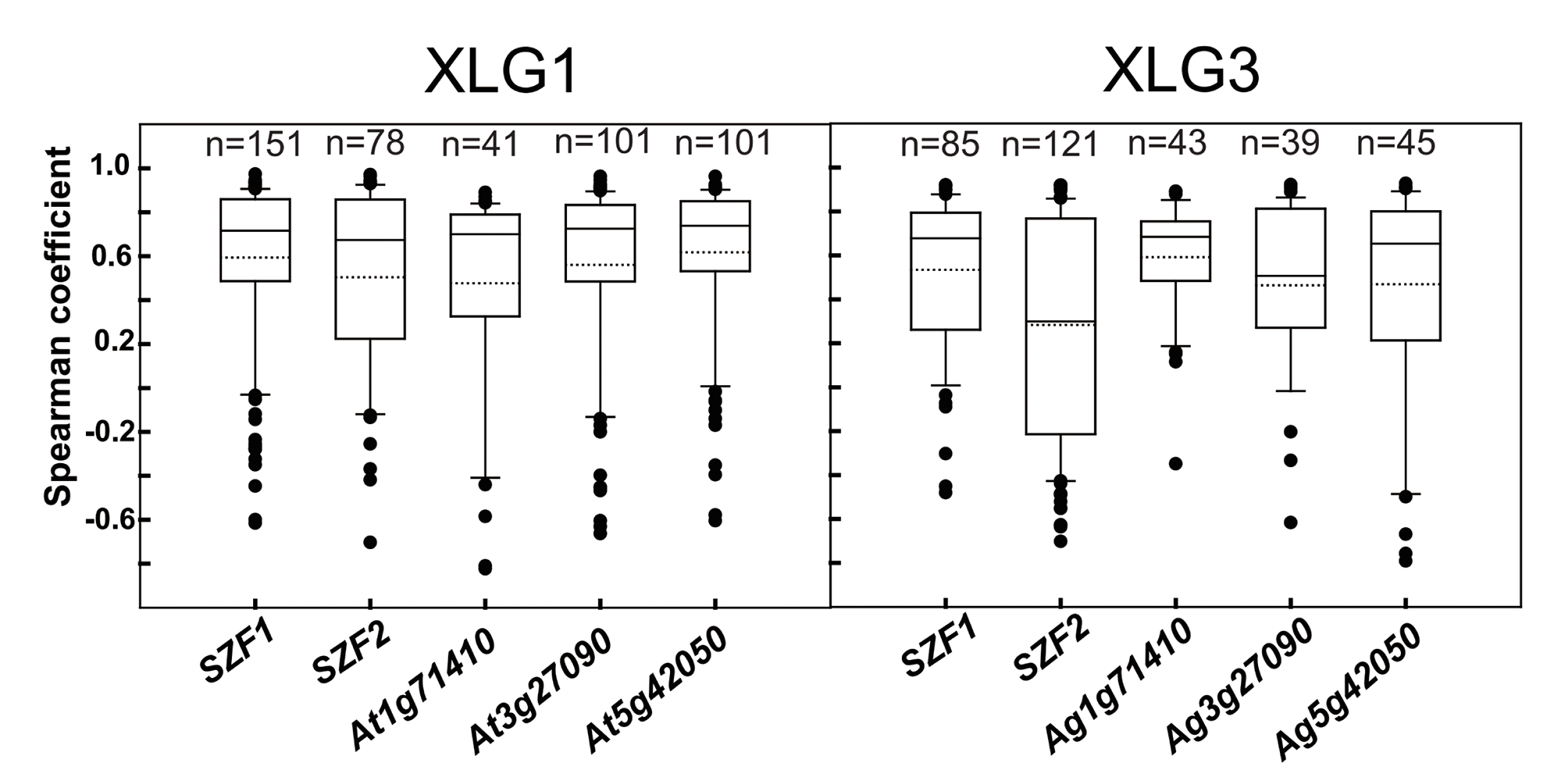

Supplement: Supplementary Figure S3 — Spearman Rank Order coefficients for nuclear localization. This coefficient is to examine the colocalization between the nucleus marker NLS-CFP and the yellow fluorescence. The range of the coefficient is from −1, a strong negative correlation, to +1, a strong positive correlation. The p-value above 0.195 (the spearman coefficient when, p < 0.05, degree of freedom = 100) is positive correlated. Box plots indicate the distribution of the coefficient and the number of the samples labeled in the figure. The solid line inside the box indicates the median and the dotted line indicates the mean value. The bottom and the top of the box represent first and third quartiles. The start and the end of the whiskers represented the maximum and minimum of the values. The dots represent the outliers. [file Image3.TIF]

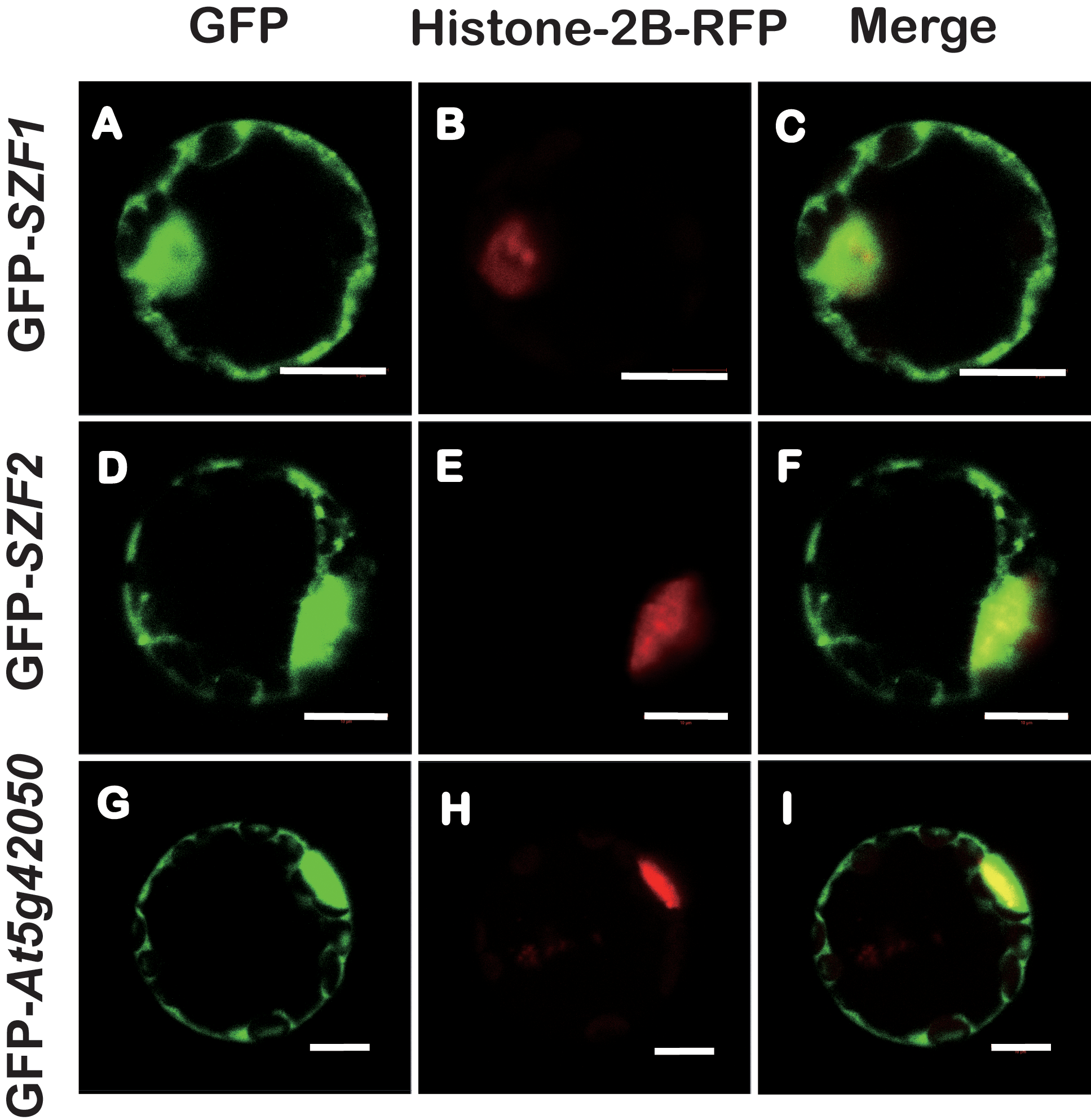

Supplement: Supplementary Figure S4 — Subcellular localization of SZF1, SZF2 and DCD protein At5g42050. Protoplasts were isolated from 5-week-old Arabidopsis Col and transformed with GFP-SZF1, GFP-SZF2 and GFP-At5g42050 in addition to the nucleus marker Histone-2B-RFP. The constructs (GFP-SZF1, GFP-SZF2 and GFP-At5g42050) are listed to the left of the images. Each construct has three panels from left to right, A, D and G, GFP signal are the expression patterns of theindicated target proteins; (B, E, and H), RFP signal is the nucleus signal; (C, F, and I), the merge images of GFP and RFP. Bar = 10 μm. [file Image4.TIF]

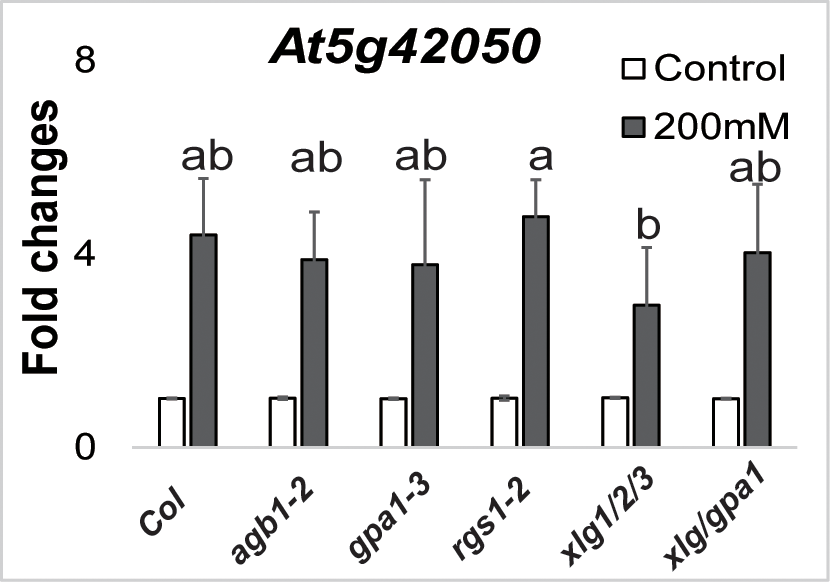

Supplement: Supplementary Figure S5 — Salt-induced DCD domain protein At5g42050 gene expression. Seven-day-old Arabidopsis seedlings Col and G protein mutants (agb1-2, gpa1-3, rgs1-2, xlg1/xlg2/xlg3 and xlg1/xlg2/xlg3/gpa1-3) grown hydroponically in dim light room were treated with 200mM NaCl for 2h. ANOVA analysis with SAS8.0, p < 0.05, five biological replicates. [file Image5.TIF]

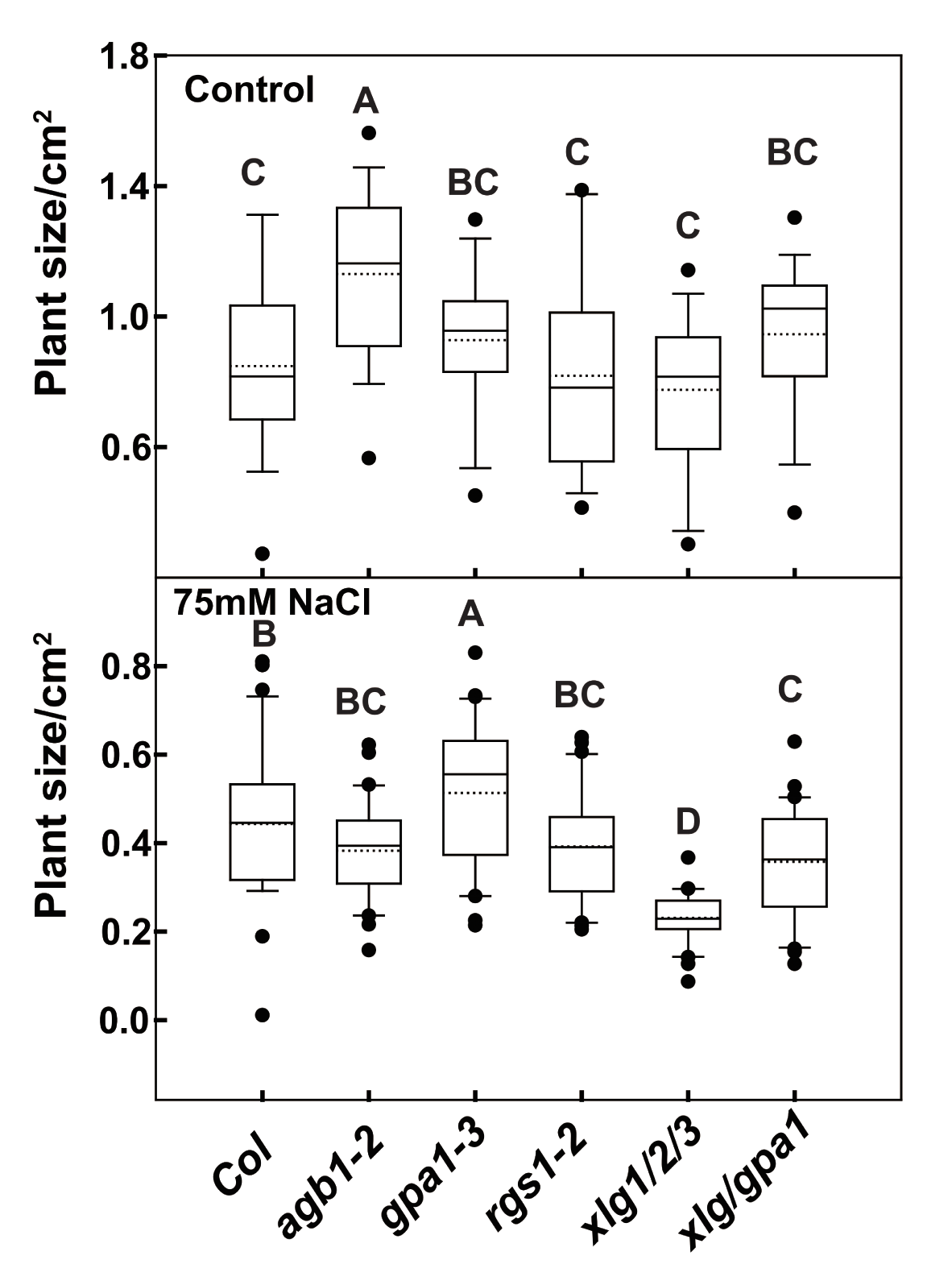

Supplement: Supplementary Figure S6 — Salt phenotypes of the G protein under mild salt stress. Seedlings of the indicated genotypes were grown in 1/2 MS medium with or without 75mM NaCl for 18 days. Box plot indicates the distribution leaf areas. The solid line in the box indicates the median and the dot line indicates the mean value. The bottom and the top of the box represent first and third quartiles. The start and the end of the whiskers represent the maximum and minimum of the values. The dots represent the outliers. Different lowercases letter indicated significant differences (p < 0.05) between any two genotypes. The ANOVA analysis with SAS8.0, n = 24. [file Image6.TIF]
