# Supplementary material for: Extra Large G-Protein Interactome Reveals Multiple Stress Response Function and Partner-Dependent XLG Subcellular Localization
Source: Front Plant Sci. 2017 Jun 13;8:1015. doi: 10.3389/fpls.2017.01015 (PMC5469152; doi:10.3389/fpls.2017.01015)
Supplement: Supplementary Table S1 — GO enrichment analysis of the interactors. This excel file provides the GO analysis results from the BiNGO. The p < 0.05, and the correction p-value was performed by Benjamini & Hochberg False Discovery Rate (FDR) correction. [file Table1.PDF]

Supplemental table S1. The GO analysis of the XLG interactors.

GO-ID	p-value	corr p-value	Description	Genes in test set
6950	2.87E-06	7.57E-04	response to stress	AT4G28610|AT3G03780|AT2G30490|AT5G16470|AT2G34930|AT1G55450|AT1G26380|AT4G02380|AT1G70200|AT3G60750|AT3G26520|AT2G30860|AT5G17920|AT1G44170|AT4G15910|AT2G21620|AT2G01140|AT2G43620|AT2G41430|AT2G40140|AT5G59880|AT3G11630
9628	1.73E-06	7.57E-04	response to abiotic stimulus	AT4G28610|AT3G03780|AT2G30490|AT1G55450|AT4G02380|AT1G70200|AT3G60750|AT5G17920|AT1G44170|AT3G19820|AT1G29930|AT4G15910|AT2G21620|AT2G41430|AT2G40140|AT3G11630
9086	2.70E-05	3.73E-03	methionine biosynthetic process	AT3G03780|AT4G19710|AT5G17920
50896	2.83E-05	3.73E-03	response to stimulus	AT4G28610|AT3G03780|AT2G30490|AT2G34930|AT3G55980|AT1G26380|AT4G02380|AT3G60750|AT3G26520|AT3G19820|AT4G15910|AT5G57740|AT2G01140|AT2G43620|AT1G75240|AT5G59880|AT5G16470|AT1G55450|AT1G70200|AT2G30860|AT5G17920|AT1G44170|AT1G29930|AT2G21620|AT2G41430|AT2G40140|AT3G11630
50667	7.40E-05	7.79E-03	homocysteine metabolic process	AT3G03780|AT5G17920
46686	1.20E-04	1.05E-02	response to cadmium ion	AT3G03780|AT2G01140|AT3G60750|AT2G30860|AT5G59880|AT5G17920
97	2.33E-04	1.25E-02	sulfur amino acid biosynthetic process	AT3G03780|AT4G19710|AT5G17920
6555	2.60E-04	1.25E-02	methionine metabolic process	AT3G03780|AT4G19710|AT5G17920
9067	2.08E-04	1.25E-02	aspartate family amino acid biosynthetic process	AT3G03780|AT4G19710|AT5G17920
10035	1.77E-04	1.25E-02	response to inorganic substance	AT3G03780|AT5G16470|AT4G02380|AT2G01140|AT3G60750|AT2G30860|AT5G59880|AT5G17920
42221	2.38E-04	1.25E-02	response to chemical stimulus	AT3G03780|AT5G16470|AT3G55980|AT1G26380|AT4G02380|AT3G60750|AT2G30860|AT5G17920|AT1G44170|AT4G15910|AT5G57740|AT2G21620|AT2G

				01140|AT2G41430|AT1G75240|AT2G40140|AT5G59880
96	9.33E-04	3.07E-02	sulfur amino acid metabolic process	AT3G03780|AT4G19710|AT5G17920
9414	8.04E-04	3.07E-02	response to water deprivation	AT1G44170|AT4G15910|AT2G21620|AT4G02380|AT2G41430
9415	8.85E-04	3.07E-02	response to water	AT1G44170|AT4G15910|AT2G21620|AT4G02380|AT2G41430
10038	7.58E-04	3.07E-02	response to metal ion	AT3G03780|AT2G01140|AT3G60750|AT2G30860|AT5G59880|AT5G17920
19253	9.18E-04	3.07E-02	reductive pentose-phosphate cycle	AT3G60750|AT2G21170
19685	1.01E-03	3.14E-02	photosynthesis, dark reaction	AT3G60750|AT2G21170
9066	1.17E-03	3.33E-02	aspartate family amino acid metabolic process	AT3G03780|AT4G19710|AT5G17920
9269	1.33E-03	3.33E-02	response to desiccation	AT1G44170|AT2G21620
15977	1.33E-03	3.33E-02	carbon fixation	AT3G60750|AT2G21170
71704	1.33E-03	3.33E-02	organic substance metabolic process	AT3G60750|AT2G21170
55114	1.43E-03	3.43E-02	oxidation reduction	AT1G44170|AT2G30490|AT3G19820|AT2G34410|AT4G19710|AT1G26380|AT5G42850|AT2G30860|AT3G11630|AT1G76160
6091	1.65E-03	3.78E-02	generation of precursor metabolites and energy	AT1G29930|AT1G54780|AT2G01140|AT2G21170|AT2G33040
15979	1.82E-03	3.99E-02	photosynthesis	AT1G29930|AT1G54780|AT3G60750|AT2G21170
6642	2.25E-03	4.73E-02	triglyceride mobilization	AT2G21170
