# Supplementary material for: Extra Large G-Protein Interactome Reveals Multiple Stress Response Function and Partner-Dependent XLG Subcellular Localization
Source: Front Plant Sci. 2017 Jun 13;8:1015. doi: 10.3389/fpls.2017.01015 (PMC5469152; doi:10.3389/fpls.2017.01015)
Supplement: Supplementary Table S2 — The primers used in this paper. Shown are the primers used for the indicated TOPO cloning and the qRT-PCR reactions. [file Table2.PDF]

Supplemental table S2. Primers used in this paper

	name	AGI number	sequence	Purpose
1	XLG1-Fw	At4g19710	CACCATGCCATTGAAAGAAGATGA	TOPO clone
2	XLG1-stop-Rv		TCAATTTGAGAAGGAACTTGGCTCA	TOPO clone
3	XLG1-No stop-Rv		ATTTGAGAAGGAACTTGGCTCAGT	TOPO clone
4	XLG2-Fw	At5g20635	CACCATGGCTGCAGTTATAAGAAA	TOPO clone
5	XLG2-stop-Rv		TCAAGAGGACGAGCTGGC CT	TOPO clone
6	XLG2-No stop-Rv		AGAGGACGAGCTGGCCTCTAT	TOPO clone
7	XLG3-Fw	At1g04040	CACCATGGAGAAGAAAGATGAAGG	TOPO clone
8	XLG3-stop-Rv		TTACTCCTCCGGTCTATATGGTG	TOPO clone
9	XLG3-No stop-Rv		CTCCTCCGGTCTATATGGTGA T	TOPO clone
10	AT1G44170-FW	At1g44170	CACCATGGCTGCGAAGAAGGTTTTTGGATCG	TOPO clone
11	AT1G44170-RV		AGCTAAACCGAGAAGGACTTTGAATAAATCGA	TOPO clone
12	AT1G71410-FW	At1g71410	CACCATGTCGATAAACATGAAAACATTTACTC	TOPO clone
13	AT1G71410-RV		TAATAG ATC CAA TAG AGA TGG TTG TG	TOPO clone
14	AT2G38480-FW	At2g38480	CACCATGACGAATCCCGATAACATGAAACCG	TOPO clone
15	AT2G38480-RV		GATAAAAGAATGAGTGGAGAGTTTGTAA	TOPO clone
16	AT3G19640-FW	At3g19640	CACCATGAGAGGAGCTAGACCCGATGAATTC	TOPO clone
17	AT3G19640-RV		TTCAAGAAGGCGCTTGTACTTGCACC	TOPO clone
18	AT4G02380-FW	At4g02380	CACCATGGCTCGTTCTATCTCTAACGTTAA	TOPO clone
19	AT4G02380-RV		CTGCTTGTTGTTCAAGAGAGCTG	TOPO clone
20	AT4G15910-FW	At4g15910	CACCATGGCCGCTCGTTCACTCTC	TOPO clone
21	AT4G15910-RV		GAAAGACTTTGCTTTGTTTTTCAAAAGCAACT	TOPO clone
22	AT4G18140-FW	At4g18140	CACCATGCCAATGCCATTTTTGAAAATGAAGAGC	TOPO clone
23	AT4G18140-RV		ATCAAAAGAACTTCTTGTTGAATGATCG	TOPO clone
24	AT5G42050-FW	At5g42050	CACCATGGAGTATAATAACAACAATCAGCAATC	TOPO clone
25	AT5G42050-RV		AGGGTTTTGGTCAGCAAAAATGTC	TOPO clone
26	AT5G44340-FW	At5g44340	CACCATGAGAGAGATCCTTCATATCCAAGGC	TOPO clone
27	AT5G44340-RV		AGTCTCGTACTCCTCTTCTTCCTCCT	TOPO clone
28	AT5G45760-FW	At5g45760	CACCATGGCTACGGCAATGGAAGAGC	TOPO clone
29	AT5G45760-RV		AGAGATTGAGTAAACCTTAACTACTCTTG	TOPO clone
30	AT1G73030-FW	At1g73030	CACCATGGGTAACACAGATAAGCTGATG	TOPO clone
31	AT1G73030-RV		TCCTCTGGCTTTAAGCTCCGC	TOPO clone
32	AT3G26520-FW	At3g26520	CACCATGCCGACCAGAAACATCGCC	TOPO clone
33	AT3G26520-RV		GTAATCGGTGGTAGGCAATTGCT	TOPO clone
34	AT3G27090-FW	At3g27090	CACCATGGACAGCTTCTGGCAATTAGG	TOPO clone
35	AT3G27090-RV		TGCAGAACCAGCTTGTTCGCAAAG	TOPO clone
36	AT3G42050-FW	At3g42050	CACCATGGATCAGGCGGAGCTGAGT	TOPO clone
37	AT3G42050-RV		AGCTTGCAAGAAGCTAGCGTACT	TOPO clone
38	AT3G60210-FW	At3g60210	CACCATGGCTTCGAGTTTCATTACAGTACC	TOPO clone
39	AT3G60210-RV		CTGGACGATAGCTAACAAGTCGCT	TOPO clone
40	AT4G09580-FW	At4g09580	CACCATGGCGGCTCCTCGGAATTTAA	TOPO clone

41	AT4G09580-RV		TTCATATACTCTCTTTCTCTTTAGAAGG	TOPO clone
42	AT4G28610-FW	At4g28610	CACCATGGAGGCTCGTCCAGTTCATA	TOPO clone
43	AT4G28610-RV		ATTATCGATTTTGGGACGCTTTGG	TOPO clone
44	AT5G66240-FW	At5g66240	CACCATGGTGGAATTTACAGAGAGAGAG	TOPO clone
45	AT5G66240-RV		TTTCCGAATGGCATAAGCTGGC	TOPO clone
46	EF-1a-RT-FW	At5g60390	TGAGCACGCTCTTCTTGCTTTCA	qPCR
47	EF-1a-RT-RV		GGTGGTGGCATCCATCTTGTTACA	qPCR
48	SZF1-RT-FW	At3g55980	CCCGGCTTTGTAAAGATGAA	qPCR
49	SZF1-RT-RV		ATTGGAGAAACCGGAGGAGT	qPCR
50	SZF2-RT-FW	At2g40140	CATCGCCAATGAACTCTCCT	qPCR
51	SZF2-RT-RV		CCAAGTCTCCGGTTATCCAA	qPCR
52	NRP1-RT-Fw	At5g42050	GCTTTTCGGATTGCCACCAAGATAC	qPCR
53	NRP1-RT-RV		CCAGTGGCAAACATACTTTCCTCGT	qPCR
