# Supplementary material for: Extra Large G-Protein Interactome Reveals Multiple Stress Response Function and Partner-Dependent XLG Subcellular Localization
Source: Front Plant Sci. 2017 Jun 13;8:1015. doi: 10.3389/fpls.2017.01015 (PMC5469152; doi:10.3389/fpls.2017.01015)
Supplement: Supplementary Table S3 — The quantitative fold changes for the real time PCR. Shown are the quantitative fold changes for the real time PCR in corresponding to Figures 4E,F and Figure S5. ANOVA analysis with SAS8.0, p < 0.05, five biological replicates. The value indicates the standard error. [file Table3.PDF]

Supplemental table 3. Relative fold changes of gene expression (mean ± s.e., n ≥ 5) in different genotype of *Arabidopsis thaliana* under Control (0 mM NaCl) and NaCl (200 mM NaCl) treatment.

Gene	treatment	Col	*agb1-2*	*gpa1-3*	*rgs1-2*	*xlg1/2/3*	*xlg/gpa1*
SZF1	Control	1.01±0.002	1.03±0.008	1.02±0.007	1.03±0.026	1.02±0.004	1.03±0.007
	200mM NaCl	7.99±0.295	5.49±0.483	12.65±1.099	11.76±1.025	4.83±0.285	10.31±0.563
SZF2	Control	1.02±0.006	1.02±0.006	1.01±0.004	1.03±0.013	1.04±0.014	1.02±0.002
	200mM NaCl	5.72±0.366	4.01±0.455	7.60±1.196	8.12±0.700	2.69±0.890	5.88±0.632
At5g42050	Control	1.01±0.002	1.02±0.011	1.01±0.006	1.02±0.017	1.03±0.008	1.01±0.004
	200mM NaCl	4.40±0.322	3.89±0.440	3.78±0.717	4.77±0.288	2.94±0.691	4.03±0.633

Note: This data is the quantitative fold changes data for Figure 4E, F and Fig.S5.
