# Supplementary material for: Extra Large G-Protein Interactome Reveals Multiple Stress Response Function and Partner-Dependent XLG Subcellular Localization
Source: Front Plant Sci. 2017 Jun 13;8:1015. doi: 10.3389/fpls.2017.01015 (PMC5469152; doi:10.3389/fpls.2017.01015)
Supplement: Supplementary Table S4 — The raw data for the Y2H. This table provides the raw data for the Y2H without filtering the published artifacts. The known artifacts were in red. [file Table4.PDF]

Table S4. The raw data for the yeast two hybrid.

Prey	Bait	[1]p value	[2]coefficient	description	library
XLG1	At1g43170	1.5E-06	0.07	60S ribosomal protein L3-1	Glucose
XLG1	At1g44170	4.4E-15	0.08	aldehyde dehydrogenase 3H1	Root
XLG1	At1g55450	5.4E-03	(0.02)	S-adenosyl-L-methionine-dependent methyltransferases superfamily protein	SAL
XLG1	At1g56070	1.7E-10	0.10	elongation factor EF-2-like protein LOS1	Glucose
XLG1	At1g75240	1.3E-32	0.07	homeobox protein 33	Glucose
XLG1	At2g21160	2.3E-23	0.08	translocon-associated protein subunit alpha	SAL
XLG1	At2g21620	3.1E-01	(0.01)	dessication responsive protein	Root
XLG1	At2g30160	2.4E-02	0.05	mitochondrial substrate carrier family protein	Glucose
XLG1	At2g34930	4.2E-02	(0.02)	disease resistance-like protein/LRR domain-containing protein	Root
XLG1	At2g44450	1.3E-04	(0.02)	beta glucosidase 15	SAL
XLG1	At3g04500	1.6E-23	0.22	RNA recognition motif-containing protein	Glucose
XLG1	At3g11630	5.5E-22	0.08	2-Cys peroxiredoxin BAS1	Glucose
XLG1	At3g55980	1.7E-03	0.02	hypothetical protein	Root
XLG1	At3g55980	9.2E-04	0.02	salt-inducible zinc finger 1	Root
XLG1	At4g02380	2.5E-01	(0.01)	senescence-associated protein SAG21	Glucose
XLG1	At4g09580	2.5E-31	0.27	SNARE associated Golgi protein family	Root
XLG1	At4g18140	5.0E-33	0.13	SCP1-like small phosphatase 4b	Root
XLG1	At4g19710	3.9E-22	0.12	bifunctional aspartokinase/homoserine dehydrogenase 2	Glucose
XLG1	At4g37910	7.5E-03	0.03	heat shock protein 70-1 m	Glucose
XLG1	At4g38770	7.2E-09	0.03	proline-rich protein 4	Glucose
XLG1	At4g39870	3.9E-15	0.15	TLD-domain containing nucleolar protein	Root
XLG1	At5g06350	1.5E-16	0.12	Rix1 complex component domain-containing protein	Glucose
XLG1	At5g12110	8.5E-02	0.01	Elongation factor 1-beta 1	SAL

Prey	Bait	[1]p value	[2]coefficient	description	library
XLG1	[3]At5g38410	1.1E-06	0.03	ribulose bisphosphate carboxylase small chain 3B	Glucose
XLG1	At5g42850	1.1E-01	0.02	thioredoxin-like protein Clot	SAL
XLG1	At5g54760	6.9E-71	0.40	translation initiation factor SUI1 family protein	SAL
XLG1	At5g57740	4.0E-01	(0.01)	E3 ubiquitin-protein ligase XBAT32	SAL
XLG1	At5g59880	3.7E-07	0.05	actin depolymerizing factor 3	SAL
XLG2	At2g41840	1.5E-04	0.14	40S ribosomal protein S2-3	SAL
XLG2	At4g40040	3.8E-32	0.34	histone H3.2	SAL
XLG2	At5g16470	8.7E-28	0.30	hypothetical protein	SAL
XLG2	At5g39740	4.0E-03	(0.05)	60S ribosomal protein L5-2	SAL
XLG2	At5g42050	0.0E+00	0.62	DCD (Development and Cell Death) domain protein	Glucose
XLG3	At1g04040	5.7E-21	(0.08)	HAD superfamily, subfamily IIIB acid phosphatase	SAL
XLG3	At1g07930	6.4E-06	0.08	elongation factor 1-alpha	SAL
XLG3	At1g26380	3.2E-26	0.10	FAD-binding and BBE domain-containing protein	Glucose
XLG3	At1g29930	5.3E-24	(0.06)	putative photosystem II type I chlorophyll a/b binding protein	Glucose
XLG3	At1g31780	1.3E-17	0.25	hypothetical protein	Glucose
XLG3	At1g54780	1.3E-27	(0.08)	hypothetical protein	Glucose
XLG3	At1g67320	1.5E-19	0.17	probable DNA primase large subunit	Glucose
XLG3	At1g70200	4.8E-08	(0.06)	RNA recognition motif-containing protein	Glucose
XLG3	At1g70770	6.4E-76	0.38	hypothetical protein	Glucose
XLG3	At1g71410	9.3E-34	0.32	putative protein kinase	Root
XLG3	At1g73030	2.7E-13	0.19	ESCRT-related protein CHMP1A	Root
XLG3	At1g76160	3.3E-01	0.01	SKU5-like 5 protein	SAL
XLG3	At1g79040	5.5E-18	(0.08)	putative photosystem II polypeptide protein	Glucose
XLG3	At2g01140	6.4E-47	0.22	fructose-bisphosphate aldolase 3	SAL
XLG3	At2g04030	6.6E-01	0.01	chloroplast heat shock protein 90	Glucose

Prey	Bait	[1]p value	[2]coefficient	description	library
XLG3	At2g04390	9.5E-45	0.28	40S ribosomal protein S17-1	SAL
XLG3	At2g04410	4.8E-10	0.14	RPM1-interacting protein 4-like protein	Glucose
XLG3	At2g21170	1.3E-34	0.25	triosephosphate isomerase	Glucose
XLG3	At2g25970	6.8E-148	0.57	hypothetical protein	Glucose
XLG3	At2g27900	2.0E-105	0.54	hypothetical protein	Glucose
XLG3	At2g30490	2.2E-04	(0.04)	trans-cinnamate 4-monooxygenase	SAL
XLG3	At2g30860	6.0E-11	0.09	glutathione S-transferase PHI 9	Glucose
XLG3	At2g30960	5.6E-91	0.34	uncharacterized protein	SAL
XLG3	At2g33040	5.8E-28	0.24	ATP synthase subunit gamma	SAL
XLG3	At2g34410			O-acetyltransferase-like protein	Glucose
XLG3	At2g38480	1.3E-12	(0.14)	CASP-like protein	Glucose
XLG3	At2g39730	7.9E-44	(0.10)	ribulose bisphosphate carboxylase/oxygenase activase	Glucose
XLG3	At2g40140	1.1E-35	0.14	zinc finger CCCH domain-containing protein 29	Glucose
XLG3	At2g41430	2.0E-32	0.16	dehydration-induced protein ERD15	Glucose
XLG3	At2g43620	3.1E-04	0.03	chitinase family protein	Glucose
XLG3	At3g02120	8.2E-04	0.04	hydroxyproline-rich glycoprotein-like protein	SAL
XLG3	At3g03780	4.8E-04	0.05	methionine synthase 2	Glucose
XLG3	At3g16420	3.7E-01	0.01	PYK10-binding protein 1	Glucose
XLG3	At3g19640	1.2E-135	0.52	magnesium transporter MRS2-3	Glucose
XLG3	At3g19820	1.7E-26	0.12	Delta(24)-sterol reductase	Glucose
XLG3	At3g26520	9.5E-16	(0.07)	aquaporin TIP1-2	Root
XLG3	At3g27090	1.4E-11	0.17	DCD (Development and Cell Death) domain protein	SAL
XLG3	At3g27850	7.5E-21	(0.10)	ribosomal protein L12-C	Glucose
XLG3	At3g42050	2.1E-21	0.27	V-type proton ATPase subunit H	Glucose
XLG3	At3g44100	1.1E-31	0.27	hypothetical protein	SAL

Prey	Bait	[1]p value	[2]coefficient	description	library
XLG3	At3g58140	1.1E-10	(0.11)	phenylalanyl-tRNA synthetase	Glucose
XLG3	At3g60210	3.8E-02	(0.05)	GroES-like family protein	Root
XLG3	At3g60750	6.2E-05	(0.05)	transketolase	Glucose
XLG3	At4g02890	1.2E-56	0.38	polyubiquitin 14	Glucose
XLG3	At4g14520			DNA-directed RNA polymerase II-like protein	Glucose
XLG3	At4g15910	4.7E-70	0.13	DROUGHT-INDUCED 21	SAL
XLG3	At4g16720	3.7E-39	0.25	putative ribosomal protein	SAL
XLG3	At4g17390	1.2E-25	0.19	60S ribosomal protein L15-2	SAL
XLG3	At4g28610	1.5E-83	0.48	phosphate starvation response 1 protein	Glucose
XLG3	At4g37180	2.3E-62	0.27	myb family transcription factor	Glucose
XLG3	At5g06310			protection of telomeres 1b	Glucose
XLG3	At5g15200	6.3E-58	0.34	40S ribosomal protein S9-1	Glucose
XLG3	At5g17670	6.4E-22	(0.12)	hydrolase-like protein	Glucose
XLG3	At5g17920	3.2E-05	0.05	5-methyltetrahydropteroyltriglutamate--homocysteine methyltransferase	Glucose
XLG3	At5g19510	1.2E-19	0.19	Elongation factor 1-beta 2	Glucose
XLG3	At5g28740	1.1E-145	0.54	tetratricopeptide repeat domain-containing protein	SAL
XLG3	At5g44340	1.8E-02	0.05	tubulin beta chain 4	Glucose
XLG3	At5g45760	9.7E-14	0.11	transducin/WD40 domain-containing protein	Glucose
XLG3	At5g66240	1.0E-97	0.52	transducin family protein / WD-40 repeat family protein	Glucose

Note: The co-expression correlation coefficient indicated the regression linear results based on 1779 arrays. The [1]co-expression correlation ranged from -1 to +1, represented the correlation direction, closer to +1, the more positive relation. The negative value indicated with parenthesis. The [2]p-value indicated the strength of the correlation. The gene names in red[3] represented the known artifacts.
